# Poly(3,4‐ethylenedioxythiophene)‐Based Neural Interfaces for Recording and Stimulation: Fundamental Aspects and In Vivo Applications

**DOI:** 10.1002/advs.202104701

**Published:** 2022-02-21

**Authors:** Michele Bianchi, Anna De Salvo, Maria Asplund, Stefano Carli, Michele Di Lauro, Andreas Schulze‐Bonhage, Thomas Stieglitz, Luciano Fadiga, Fabio Biscarini

**Affiliations:** ^1^ Center for Translational Neurophysiology of Speech and Communication Fondazione Istituto Italiano di Tecnologia via Fossato di Mortara 17 Ferrara 44121 Italy; ^2^ Sezione di Fisiologia Università di Ferrara via Fossato di Mortara 17 Ferrara 44121 Italy; ^3^ Division of Nursing and Medical Technology Luleå University of Technology Luleå 971 87 Sweden; ^4^ Department of Microsystems Engineering‐IMTEK University of Freiburg Freiburg 79110 Germany; ^5^ BrainLinks‐BrainTools Center University of Freiburg Freiburg 79110 Germany; ^6^ Epilepsy Center Faculty of Medicine University of Freiburg Freiburg 79110 Germany; ^7^ Life Science Department Università di Modena e Reggio Emilia Via Campi 103 Modena 41125 Italy; ^8^ Present address: Department of Environmental and Prevention Sciences Università di Ferrara Ferrara 44121 Italy

**Keywords:** drug delivery, neural recording, organic electrochemical transistors, poly(3,4‐ethylenedioxythiophene), stimulation

## Abstract

Next‐generation neural interfaces for bidirectional communication with the central nervous system aim to achieve the intimate integration with the neural tissue with minimal neuroinflammatory response, high spatio‐temporal resolution, very high sensitivity, and readout stability. The design and manufacturing of devices for low power/low noise neural recording and safe and energy‐efficient stimulation that are, at the same time, conformable to the brain, with matched mechanical properties and biocompatibility, is a convergence area of research where neuroscientists, materials scientists, and nanotechnologists operate synergically. The biotic–abiotic neural interface, however, remains a formidable challenge that prompts for new materials platforms and innovation in device layouts. Conductive polymers (CP) are attractive materials to be interfaced with the neural tissue and to be used as sensing/stimulating electrodes because of their mixed ionic‐electronic conductivity, their low contact impedance, high charge storage capacitance, chemical versatility, and biocompatibility. This manuscript reviews the state‐of‐the‐art of poly(3,4‐ethylenedioxythiophene)‐based neural interfaces for extracellular recording and stimulation, focusing on those technological approaches that are successfully demonstrated in vivo. The aim is to highlight the most reliable and ready‐for‐clinical‐use solutions, in terms of materials technology and recording performance, other than spot major limitations and identify future trends in this field.

## Graphical abstract

A comprehensive review of the latest advances in the field of implantable neural interfaces based on poly(3,4‐ethylenedioxythiophene) and its derivates. The aim is to highlight the most reliable, translatable (or ready) solutions, in terms of material technology and recording/stimulation performances, as well as, to identify current bottlenecks and future trends of this rapidly evolving field.

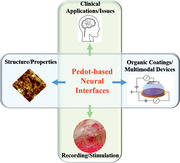

## 1 Neural Interfaces for Extracellular Recording and Stimulation

**Figure 1:**
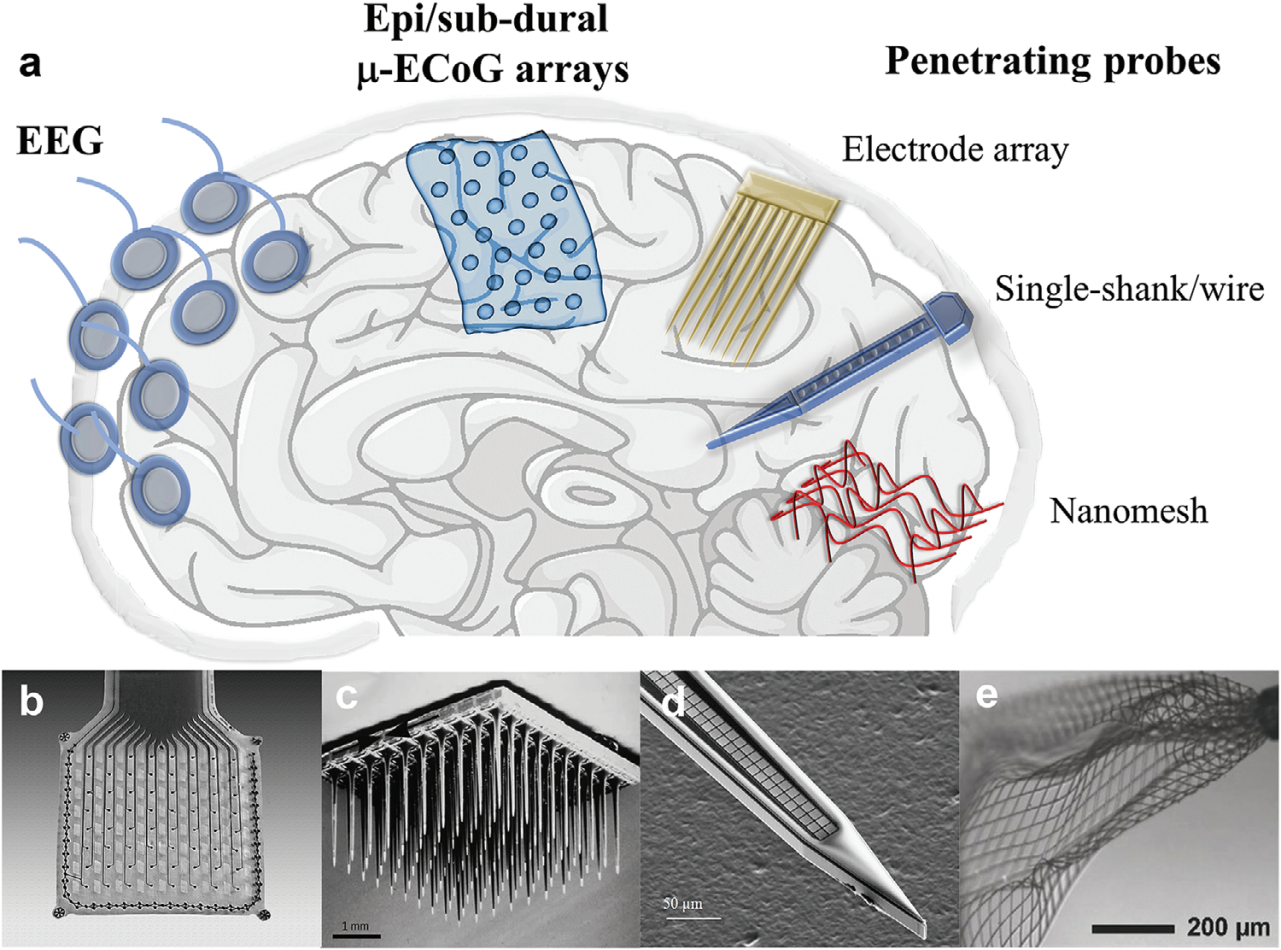
Neural devices for extracellular recording and stimulation. a) Different brain signals can be recorded through non‐invasive or invasive approaches. EEG signals are recorded from the scalp; LFP signals can be recorded from  *μ*‐ECoG arrays placed on the cortex surface. b) MuSa array intended for human studies (courtesy of Prof. Thomas Stieglitz, University of Freiburg). Single‐unit spikes are commonly recorded by penetrating neural devices such as c) microelectrode arrays (Utah array), d) single‐shank. c,d) Reproduced with permission.^[^ ^8^ ^]^ Copyright 2017, Springer Nature. e) Injectable nanomesh devices. e) Reproduced with permission.^[^ ^9^ ^]^ Copyright 2018, John Wiley and Sons.

One of the main technological challenges is the design of brain interfaces suitable to establish an effective bidirectional communication with the central nervous system (CNS) by enabling in situ transduction, modulation, and decoding of neural signals in a unique, not distributed, “resident” volume of the brain.^[^ ^1^, ^2^, ^3^, ^4^, ^5^, ^6^ ^]^ Reproducing these functions with a device would dramatically change the way we conceive brain‐machine interfaces aimed at improving patients’ quality of life, paving the way for novel therapeutic routes based on controlled stimulation, inhibition, repair or replacement of neural circuits.^[^ ^7^ ^]^ Different methods are available for recording and stimulating neuronal activity, which differ in terms of spatial/temporal resolution of the signal and device invasiveness (**Figure** **1**a). All neural devices approaches have to fulfill the requirements essential requirements on safety and functionality which include as key elements chemical and structural biocompatibility. Surface biocompatibility describes the chemical interaction (toxicity of material and eluates) with the environment while structural biocompatibility summarizes all interactions and consequences of mechanical nature that originate from a mismatch between the mechanical properties of the implant and the target tissue. Not only genuine material properties like the Young's modulus have to be taken into account but also system aspects of chronic implants like connectors, interconnection cables, shape of the probe which determines the moment of inertia and resulting (micro‐)motions during implantation. Functionally relevant information can be obtained by means of large electrodes placed on the scalp, resulting in a non‐invasive technique called electroencephalography (EEG) (**Table** **1**). However, EEG (but the same applies to magnetoelectroencephalography) provides rather weak and noisy electrical signals that generally encompass the activity of extended brain areas^[^ ^10^ ^]^ or reflect functional brain networks.^[^ ^13^ ^]^ The detection with EEG of high frequency activity (>70 Hz) is also hampered by the attenuation/filtering phenomena produced by the scalp and the skull, interposed between the electrodes and the brain. Therefore, more invasive devices such as ultra‐conformable electrocorticography (ECoG) and *μ*‐ECoG (Figure 1b) arrays, placed on the surface of the CNS, or intracortical probes (Figure 1c–e) should be adopted.^[^ ^8^, ^9^, ^14^, ^15^, ^16^, ^17^ ^]^ The possibility to deliver electrical recording and stimulation by endo‐ and intravascular microelectrodes has been also reported.^[^ ^11^, ^12^ ^]^ Currently, extracellular electrodes are the most viable interfaces to record local field potentials (LFPs) at high spatial‐temporal resolution and high signal‐to‐noise ratio (SNR),^[^ ^23^ ^]^ action potentials (spikes) from a population of nearest neurons ("multi‐unit" recording), and in some instances also from single neurons (single‐unit activity).^[^ ^13^, ^24^, ^25^ ^]^

**Table 1:** Clinical applications of brain recording

Site	Form	Material	El. Size	Signals	Recording sample	Clinical application	Ref.
Scalp	Cup electrodes	Ag/AgCl	10 mm/314 mm^2^/3.1 cm^2^	EEG	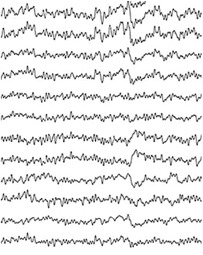	Diagnosis of epilepsy and of regional dysfunctions	^[^ ^26^ ^]^
Sub‐cutaneous	Needle electrodes	Pt‐Ir	35 mm^2^	EEG		Long‐term monitoring of seizures and interictal epileptic activity	^[^ ^27^ ^]^
Subdural	Grid electrodes, stripe electrodes	Pt‐Ir/Steel	4.15 mm^2^	LFP	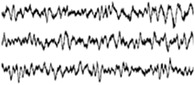	Presurgical mapping and localization of the epileptogenic region	^[^ ^32^ ^]^
	Cylinder‐shaped multicontact electrodes	Pt‐Ir/Steel	5.0 mm^2^/8.1 mm^2^	LFP		Presurgical localization of the epileptogenic region	^[^ ^31^ ^]^
Intra‐cerebral	Hybrid electrodes with microwires	Pt‐Ir	Wire: 3000 µm^3^	LFP, unit activity	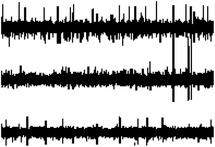	Studies in cognition and neurophysiology	^[^ ^25^, ^31^, ^32^ ^]^
	Hybrid electrodes with shafts tetrodes	Pt‐Ir					

Spikes are usually recorded by the means of penetrating probes; however, it is also possible to detect them also by the surface of the cortex using ultra‐conformable *μ*‐ECoG arrays, as it has been recently demonstrated.^[^ ^17^, ^18^, ^19^ ^]^ In addition, integrated stimulation allows to assess anatomical and functional connectivity at diverse scales of interactions.^[^ ^21^, ^22^ ^]^

Neural stimulation means the injection of current into tissue with the purpose of triggering a specific neural response. When the stimulus reaches over a certain threshold, the impulse can trigger action potentials in neurons nearby, meaning the artificially generated stimulation is translated to a real neural signal in the nervous system. The typical stimulus is a square current pulse of a few hundred μ‐seconds duration or, using common terminology, the pulse would be one “phase” of the stimulation. The threshold necessary to elicit an action potential would in this case be defined by the current amplitude. As the threshold, in turn, is linked to the duration of the pulse, so that longer pulses require lower current, it is common to note charge/phase instead of current to define the stimulation. In order to prevent build‐up of electrochemical reactions by‐products at the interface, it is common to stimulate in bi‐phasic manner, meaning the depolarizing impulse (usually cathodic) is immediately followed by the opposite pulse, retrieving the injected charge. The net‐charge transferred over such a bi‐phasic pulse will be zero, yet the outcome on the stimulated neural structure will be evoking an action potential. Stimulation pulse parameters vary depending on the adopted electrode materials^[^ ^33^ ^]^ and must be safe with respect to the electrodes, as well as, the neural target tissue.^[^ ^34^ ^]^ Simple rules of thumb to limit biological damage have been derived based on retrospective data.^[^ ^28^ ^]^ The Shannon equation^[^ ^28^ ^]^ has been proven useful for the analysis of these retrospective data and for macroelectrodes^[^ ^29^ ^]^ but has its limitations to macroelectrodes. Studies with microelectrodes that meet clinical needs but exceed conservative values of charge density and charge per phase show that these higher values do not cause tissue damage when certain boundary conditions are met.^[^ ^30^ ^]^ In addition to action potentials, stimulation can be used to influence the state of the whole system. Such neuromodulation typically employs signals with slower dynamics than the bi‐phasic pulses described above, as the intention is not eliciting action potentials in single neurons, instead to influence the network on system level (**Table** **2**).^[^ ^35^, ^36^, ^37^, ^38^, ^39^, ^40^, ^41^, ^42^, ^43^, ^44^, ^45^, ^46^ ^]^ Common terms to describe the signals are alternating current stimulation (ACS), typically a sinusoidal signal in the 0.1–200 Hz range, and direct current stimulation (DCS). For the latter, the DC stimulation is commonly “dosed” in asymmetric pulses, but with pulse lengths closer to the seconds to minute range, than the sub‐millisecond. It is important to note that even if this broad variety of pulse types, from biphasic to DC, is clinically relevant and grouped under the general term “neurostimulation,” these pulses pose very different challenges from the perspective of the electrode material. Short bi‐phasic pulses have the advantage that it is possible to inject charge mainly via capacitive mechanisms, meaning the reaction kinetics is perfectly symmetric in the anodic and cathodic phase. Longer pulses are only possible by engaging additional faradaic charge injection mechanisms on the surface, and therefore put the electrodes at higher risk for corrosion or similar electrochemically driven degradation.

**Table 2:** Clinical applications of stimulating electrodes

Mode	Pulse type and parameters	Localization	Mechanism of action	Clinical application	Ref.
DC stimulation	DC/pseudo DC 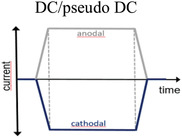	Transcranial 1–4 mA, 20 min day^−1^	Neuronal de‐/hyperpolarization	Cognitive enhancement	^[^ ^35^ ^]^
				Depression	^[^ ^36^ ^]^
				Focal epilepsy	^[^ ^37^, ^38^ ^]^
Low frequency stimulation (<10 Hz)	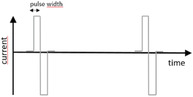	Transcranial: 0.5–10 Hz, 1–4 mA Subcortical: 2–4 Hz	Induction of long‐term depression (LTD)	Focal epilepsy (EASEE‐Device)	^[^ ^38^ ^]^
				Focal epilepsy	^[^ ^39^, ^40^ ^]^
High frequency stimulation (>100 Hz)	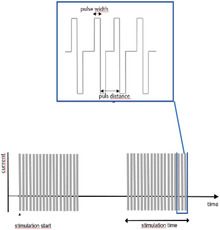	Transcranial 100–130 Hz, 1–12 mA	Activation of hypoactive brain areas. Interference with ictal pattern generation	Depression	^[^ ^36^ ^]^
				Focal epilepsy	^[^ ^38^ ^]^
		Epicortical 30–90 Hz 100/200 Hz, amplitude 80% motor threshold	Gating modulation Interference with ictal pattern generation	Pain	^[^ ^41^ ^]^
				Focal epilepsy (RNS‐Device)	^[^ ^42^ ^]^
		Intracortical 30–40 Hz/130 Hz, 2–5 V	Targeted neurotransmitter release. Reduction of network recruitability/Interference with ictal pattern generation	Pain	^[^ ^41^ ^]^
				Psychiatric disorders	^[^ ^43^ ^]^
				Focal epilepsy	^[^ ^42^, ^44^ ^]^
Patterned stimulation (e.g., theta burst stimulation)	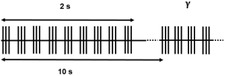	Transcranial theta 5 Hz, amplitude: 80% motor threshold Intracortical theta 4–5 Hz, 4–5 mA	Activation of physiological patterns in memory formation and spatial navigation	Cognitive enhancement	^[^ ^45^, ^46^ ^]^

### 1.1 Main Issues of Implantable Neural Interfaces

A major issue with invasive neural interfaces (more relevant for intracortical probes) is the deterioration with time of the recording/stimulation performance due to the occurring of the “foreign body reaction” (FBR),^[^ ^47^, ^48^, ^49^ ^]^ eventually leading to the encapsulation of the device by an insulating layer mainly composed of activated glia and astrocyte cells (see **Box 1**). The formation of the so‐called “glial scar,” together with concurrent reduction in the number of neurons in the proximity of the probe (within a radius of ≈100 µm)^[^ ^47^ ^]^ due to cell death or migration, deplete the amplitude of the recorded signals within a few weeks.^[^ ^50^, ^51^ ^]^ Apart from biotic factors, the quality of recording can be compromised also by abiotic mechanisms. These abiotic mechanisms are generally summarized under the technical term of “structural biocompatibility." They include cyclic stresses applied to the brain tissue due to shear micro‐motion at the interface between the device or the (stiff) connector and the (soft) brain tissue due to brain pulsations and micro‐movements,^[^ ^52^ ^]^ inability of the device to conform to the cortex surface (in particular for epicortical or subdural arrays) and delamination or chemical leaching from the electrode surface especially when coatings are applied.^[^ ^8^, ^53^ ^]^ In order to limit the impact of the FBR and maintain stable, high quality chronic recording and stimulation, technological advances are oriented toward the manufacturing of highly‐flexible compliant devices^[^ ^16^ ^]^ and minimally‐invasive probes such as penetrating carbon microfibers^[^ ^54^ ^]^ or injectable nanomesh probes^[^ ^55^, ^56^ ^]^ (Figure 1e). In this regard, it was demonstrated that implants with lateral size <10 micron (i.e., corresponding to the average size of a neuron soma) cause an almost negligible inflammatory response, enabling stable high‐quality recording in chronic scenarios.^[^ ^57^ ^]^ This can be ascribed to reduced tissue displacement (intracortical probes) or depression (epicortical arrays), limited blood‐brain‐barrier (BBB) disruption and vasogenic edema, and minimally impaired transport of signaling molecules.^[^ ^57^ ^]^ Besides, in order to minimize the mechanical mismatch at the nervous tissue/neural device interface, materials research is devoted to the design of neural devices using flexible (polyimide PI, parylene PAR), or ultra‐flexible (e.g., polydimethylsiloxane (PDMS), hydrogels) materials, showing Young's modulus from ≈6 orders of magnitude larger to similar values to that of the brain tissue (0.01–10 kPa), respectively.^[^ ^58^, ^59^ ^]^ However, it is worthwhile reminding that the combination of modulus and device geometry/cross‐section (which is the device's moment of inertia) confers high compliance to the device. It is indeed possible to create highly flexible probes and surface arrays with relatively stiff materials showing bending stiffness comparable to that of brain tissue (0.1–100 pN^.^ m)^[^ ^16^ ^]^ leading to only minor chronic tissue shears and negligible upregulation of inflammatory cytokines.^[^ ^55^, ^60^ ^]^ These considerations are especially useful when considering conjugated materials and specifically conductive polymers, as it will be discussed in detail below.

**Box 1. Neural Tissue Reaction to Probe Implantation**

**Figure B1:**
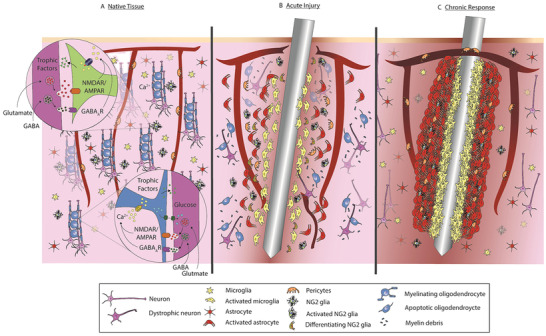
Invasive devices elicit a localized immune response. a) Cellular components in healthy brain tissue. Axons signal both oligodendrocytes and NG2 glia and neuronal modulation via glutamatergic and GABAergic neurotransmitter release. b) Acute injury after device insertion. Disrupted blood vessels leak inflammatory factors. Microglia, astrocytes, and NG2 glia become activated and adhere to the surface of the electrode. c) Chronic immune response to implanted devices. Glial cells form a chemical and mechanical barrier around the device, preventing the transmission of ions, charged solutes, and neurochemical signals, compromising the quality of signal recording. Reproduced with permission.^[^ ^63^ ^]^ Copyright 2017, American Chemical Society.

Breaching of the BBB and of cell membrane, mechanical strain, and inflammation upon probe implantation, are regarded as the first steps of the biochemical signaling cascade of events targeting a variety of cell types (**Figure** **B1**). The inflammatory cascade leads to acute and later on chronic host tissue response, that eventually lowers the quality of recording, and may cause implant mobilization and failure.^[^ ^60^ ^]^ Upon BBB rupture, blood‐serum proteins (such as albumin, globulins, fibrin/fibrinogen), blood cells, pro‐inflammatory molecules, and cytokines are spilled in the brain parenchyma around the neural devices, where they activate the inflammatory pathways of nearby microglia and astrocytes. Activated microglia and monocytes start to encapsulate implant with lamellipodia within the first hour from implantation; during the next 12 h activated microglia become motile and move toward the injury site where they complete the formation of a thin encapsulation sheath.^[^ ^60^, ^61^ ^]^ Simultaneously, the upregulation of pro‐inflammatory cytokines drives nearby neurons toward excitotoxicity and neurodegeneration. Tight junctions among glial cells limit ionic exchange with the electrode surface, giving rise to the dramatic increase of the electrochemical impedance usually observed during the first week from implantation.^[^ ^62^, ^63^ ^]^

Astrocytes, the most abundant cells in brain providing metabolic support to neurons and guaranteeing processing and transfer of synaptic information across neuronal networks,^[^ ^64^ ^]^ are also highly activated during the first week and give rise to a compact layer around the implant during week 2 and 3,^[^ ^54^ ^]^ further worsening the recording performance. Other than microglia and astrocytes, non‐neuronal cells such as, pericytes, NG2‐glia (oligodendrocyte precursors), monocytes, and leukocytes (infiltrating upon BBB disruption) were demonstrated to participate to the gliosis formation process.^[^ ^61^, ^65^, ^66^ ^]^ The expression of cytokines and chemokines is a hallmark of chronic inflammatory response. Cytokine production can begin to occur as early as day 1; for example, acute inflammation at 0–3 days involves TNF*α*, IL‐1*β*, and IL‐6 while early chronic tissue response (3–7 days) may involve IL‐10, TGF‐*β*, and PDGF. The gliosis process is generally assumed to reach a steady state 3 to 6 months post‐implantation, greatly affecting both extracellular recording and neurostimulation quality and efficacy.^[^ ^58^, ^67^ ^]^

**Box 2. The Electrochemical Tissue‐Electrode Interface**

**Figure B2:**
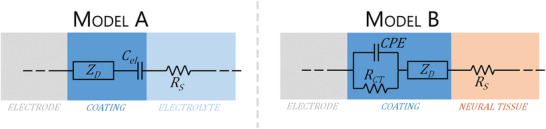
Typical equivalent circuit models adopted to describe the electrochemical impedance interface for PEDOT/PSS coated microelectrodes during in vitro (model A), or in vivo (model B) studies.

Electrochemical impedance spectroscopy (EIS) represents one of the most important tool of analysis since it is known that neural biological activity exhibits a characteristic frequency of 1 kHz thus, the impedance values at 1 kHz are often related to the high quality of a neural electrode.^[^ ^33^ ^]^ Nevertheless, impedance data are preferably collected over a wide range of frequencies, typically from 10 kHz down to below 1 Hz, in order to gain insights also on the charge transfer dynamics along the conductive films due to the diffusion of ions.^[^ ^103^ ^]^ In general, a dramatic decrease of the impedance, especially over the low frequency domain, represents the typical footprint of a conductive polymer film efficiently deposited on a metal‐based electrode. This is due to the fact that CPs exhibit both electronic, as well as, ionic conductance, with respect to metal based electrodes, thereby enabling a dramatic reduction of the imaginary part of the impedance *Z*_I_, which can be expressed as: *Z*_I_ = −1/(2*π × f × C*_dl_), where *f* is the frequency and *C*_dl_ is the double layer capacitance. One of the first studies of PEDOT/PSS electrodeposited on neural microelectrodes was reported by D. Martin et al. in 2003.^[^ ^154^ ^]^ They showed how PEDOT:PSS coating can decrease the impedance modulus |Z| by almost two orders of magnitude when compared to the uncoated metal electrodes. It was also reported that small gold microelectrodes (15 µm in diameter) coated with PEDOT:PSS were able to record higher‐quality neural activity and exhibited a much higher SNR than uncoated Au electrodes.^[^ ^155^ ^]^ To understand the effect of PEDOT:PSS on the SNR, a brief digression on the possible sources of (biological and non‐biological) noise is necessary. Non‐biological sources include electronic noise from the amplifying circuit, the injection noise at the electrolyte‐electrode interface, and the thermal noise, which is the major contribution correlated to the electrochemical properties of the coating. Thermal noise ($V_{\mathrm{rms}}^{\mathrm{th}}$) is directly related to the real part of the impedance *Z*_R_ = Re{Z} of the recording microelectrode and can be expressed as:

(1) $$V_{\mathrm{rms}}^{\mathrm{th}}=\;\sqrt{4k_{b}TZ_{R}\Delta f}$$

where *k*_b_ is Boltzmann constant, *T* is the absolute temperature, and Δ*f* is the recording bandwidth. To explain the role of the electrode interface on *Z*_R_, we discuss the widely adopted equivalent circuit consisting of the solution resistance *R*_s_ connected in series with an electronic capacitance *C*_el_, which is linked to the space charge of conductive film, and a finite‐length Warburg impedance *Z*_D_, as depicted in **Figure** **B2** – **model A**.^[^ ^154^, ^156^ ^]^

The Warburg impedance Z_D_ accounts for ion diffusion through PEDOT:PSS pores and can be expressed by:

(2) $$Z_{D}=R_{D}\;\frac{\coth\sqrt{j2\pi f\tau_{D}}}{\sqrt{j2\pi f\tau_{D}}}$$

where *R*_D_ is the diffusion resistance, *j* is the imaginary unit (−*j* ^2^ = 1), and *τ*_D_ is the ion diffusion characteristic time.^[^ ^157^ ^]^ The *Z*_D_ element is related to the diffusional pseudo‐capacitance *C*_D_, according to the formula *τ*_D_ = *R*_D_ *C*_D_. The leading terms in the expansion of Equation (2) for  *fτ*_D_ → 0 are Re{*Z*_D_} = *R*_D_/3 and Im{*Z*_D_} = −1/(2*π × f × C_D_*). In general, the Re{*Z*} is frequency independent in the case of PEDOT/PSS coatings, unlike the uncoated and flat electrodes,^[^ ^33^ ^]^ and the resulting impedance plot is dominated by a resistive behavior in the high frequencies, and a capacitance contribution in the middle to low frequency domain.

In addition, larger electroactive surface area (ESA), if compared to flat and non‐porous metal electrodes, reduces *R*_S_ = *ρ* l/ESA where *ρ* is the solution resistivity and is the electrode distance.^[^ ^77^ ^]^ Thus, altogether this results in lower Re{*Z*} values for PEDOT/PSS coated microelectrodes, so it is advantageous to produce a decrease in thermal noise. As far as the capacitance of PEDOT/PSS is concerned, according to model A of Figure B2, it can be expressed as

(3) $$C_{\mathrm{dl}}={\left[1/C_{D}+1/C_{\mathrm{el}}\right]}^{-1}\;$$

Its porous structure enables fast and efficient ion diffusion inside the material, thus yielding larger *C*_dl_ if compared to flat metal electrodes. This improvement results highly effective in reducing the dangerous polarization voltages which are typically reached upon fast current pulses adopted during neural stimulating protocols. When PEDOT:PSS coated microelectrodes are used for in vivo experiments, a further element should be added to the above mentioned circuit model. This is due to the intimate interaction between the neural microelectrode and the surrounding inflamed neural tissue which gives rise to tissue encapsulation and gliosis. For example, the circuit model adopted by Cui et al. (Figure B2 – model B) to describe the impedance traces for in vivo electrochemical analysis with PEDOT coated neural microelectrodes, consisted of a solution resistance in series with the above described Warburg element and a charge transfer element (a charge transfer resistance *R*_CT_ in parallel with a constant phase element). Interestingly, the magnitude of the charge transfer resistance can be used as a parameter to describe the extent of the neural probe encapsulated by the surrounding tissue.^[^ ^158^ ^]^

To sum up, model A represents the typical scenario that occurs for in vitro studies, when the relatively simple interface of PEDOT:PSS||electrolyte can be fitted with the equivalent RCT. Besides, model B describes the typical situation for an in vivo experimental EIS, where PEDOT/PSS interacts non only with a water based electrolyte solution but also with a surrounding neural tissue, which is also biologically reactive.

### 1.2 Toward High Spatio‐Temporal Resolution

Microelectrode miniaturization and fabrication of high‐density micro‐electrocorticography (*μ*‐ECoG) arrays allow neuroscientists to record with unprecedented spatial and temporal resolution the orchestrated/synchronized activity underlying neural networks functions, as well as, to precisely stimulate selected neuronal ensembles.^[^ ^68^, ^69^, ^70^ ^]^ Nevertheless, smaller microelectrodes exhibit higher electrolyte resistance and lower double layer capacitance compared to larger microelectrodes, which results in higher impedance.^[^ ^33^, ^57^ ^]^ Since thermal noise, one of the main sources of noise during recording, scales with the electrode impedance (see **Box 2**), the output SNR is expected to be reduced upon decreasing the electrode size.^[^ ^71^, ^72^ ^]^ However, signal loss through shunt pathways as well as electronic noise due to the amplifier and flicker noise may greatly affect the SNR in vivo under certain circumstances and recording setups.^[^ ^73^ ^]^ Other issues that arise as a consequence of electrode miniaturization might be the electrical coupling between adjacent lines (crosstalk),^[^ ^74^ ^]^ the chemical instability of the electrode surface and integration hurdles due to high density small connectors and leads.^[^ ^75^, ^76^ ^]^ In view of these arguments, efforts are devoted to the modification/functionalization of the microelectrode surface with the aim to lower the overall impedance and achieve high‐quality recordings especially in case of high‐density small recording sites. In this framework, one of the most investigated approaches is to increase the roughness of the electrode surface, to increase its electroactive area and lower its impedance. In this way, flake‐like gold or nanostructured platinum electrodes were realized, showing order of magnitude lower impedance values than pristine smooth electrodes.^[^ ^77^, ^78^, ^79^ ^]^ Alternatively, smooth or porous iron oxide layers can be grown on the electrode surface by electrochemical oxidation or sputtering, respectively.^[^ ^80^ ^]^ However, activated metal oxide layers can change their chemistry and passivate with time in chronic experiments, leading to the deterioration of recording capability over time.^[^ ^33^ ^]^ In addition, the release of potentially harmful metal ions in the peri‐implant environment by electrochemical processes has not been deeply investigated so far.^[^ ^50^, ^53^, ^81^ ^]^

In the case of stimulation, the electrodes are sufficiently small and close enough to the target structure, it is possible to selectively stimulate even down to the level of a single neurons in a system, paving the way for highly selective neuromodulation applications.^[^ ^82^ ^]^ However, smaller electrodes, exhibiting high electrochemical impedance, enhance the risk for electrochemical side‐reactions as higher voltages are needed to drive a certain current, eventually exceeding the “water window.” Besides, high voltage transient can trigger the occurring of stimulation artefacts in recordings immediately following an injecting pulse.^[^ ^83^ ^]^

In this complex scenario, where mechanical, chemical, electrochemical, and biological aspects are intertwined, the choice of materials for fabricating the electrodes is extremely challenging because it is unlikely that one material encompasses all desirable properties. In the past two decades, conductive polymers (CPs) emerged as an attractive class of materials due to their highly porous structure contributing a large electroactive area, low impedance, mixed ionic‐electronic conduction, high charge storage capacitance (CSC), other biocompatibility, and possibility of bio‐functionalization. Therefore, a great deal of interest has been generated around CPs in the neuroelectronics community, driven by the prospect of achieving high performance neural devices for recording and stimulating.

## 2 Conductive Polymers

### 2.1 Properties and Advantages of Conductive Polymers

**Figure 2:**
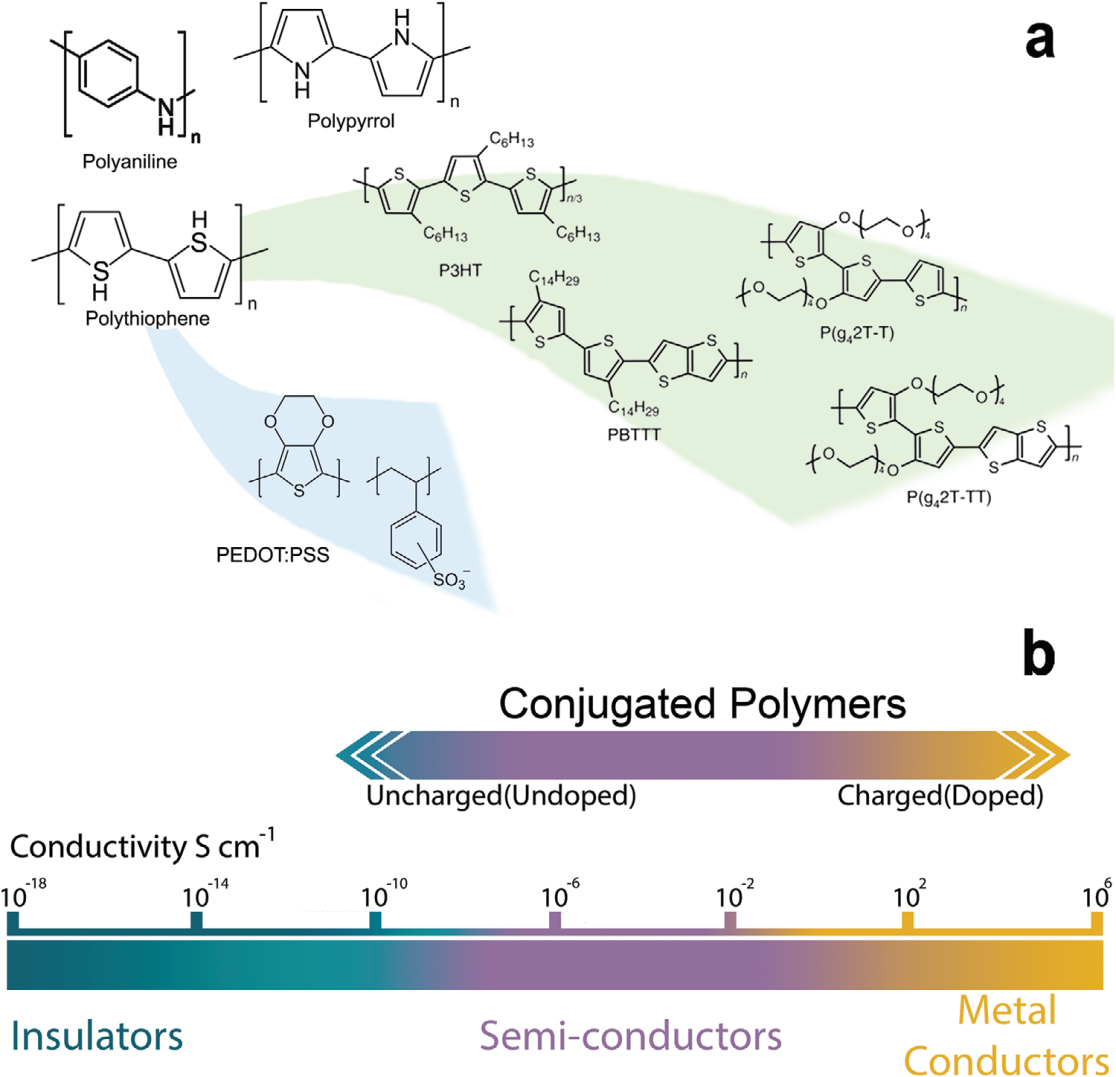
Organic (semi‐)conductors for neural applications. a) In the top left, polyaniline (PA), polypyrrole (PPy), and polythiophene (PT), three of the most common CPs for neural applications and progenitors of state‐of‐the‐art materials. Modern derivatives of polythiophene are also shown, including the widely investigated PEDOT:PSS (bottom, blue background) and a family of variously engineered polythiophenes (center/right, green background). b) Sketch of the conductivity range encompassed by (undoped and doped) conjugated polymers.

Conductive polymers (CPs), represent a versatile class of materials suitable for a wide range of applications, from microelectronics industry (including solar cells, antistatic coatings, energy storage technology)^[^ ^84^, ^85^ ^]^ up to biomedical applications.^[^ ^86^ ^]^ CPs are conjugated polymers that upon doping exhibit conductivity exceeding 1000 S cm^−1^, that is, comparable to that of metals or highly doped inorganic semiconductors (**Figure** **2**).

They are characterized by a system of extensively delocalized *π*‐molecular orbitals, and due to delocalization, charge‐separated states of these molecules (i.e., their oxidized or reduced form) are stable and long‐living. These features make the charged states to behave as “quasi‐particles,” as they possess the ability to drift and diffuse across the material. For these reasons, these charged states are also termed charge‐carriers. CPs are obtained via oxidative coupling of the monomers either by chemical synthesis (condensation or addition polymerization reactions), electrochemical polymerization, or photo‐induced oxidation.^[^ ^87^ ^]^ As in organic semiconductors, charge transport in CPs occurs via hopping between localized Wannier states, conversely to what happens in metals, in which electrons are free to occupy any position in the conduction band. The term “doping,” lexically borrowed by the semiconductor field, refers to the generation of charge carriers in the CPs, either via injection of extra electrons (i.e., reduction or n‐type doping) or removal of electrons, creating so‐called “holes‟ (i.e., oxidation or p‐type doping) that can be transported across the conjugated polymer backbone upon application of an external bias field. In the simplest and most common scenario, that is, conduction in a p‐type CP film between two electrodes upon application of a bias, holes are injected (electrons are removed) at the positive electrode and are collected at the negative one, in a cascade of localized oxidation reactions that proceed from the positive to the negative electrode. Doping can be performed either chemically or electrochemically^[^ ^88^ ^]^ during CP synthesis, generating charge carriers that are usually in the form of polarons (i.e., radical anions/cations) or bipolarons (i.e., dianions/dications). During device operation it is possible to electrostatically control the amount of mobile charge carriers, allowing switching between insulating and conductive regimes.^[^ ^89^ ^]^ The conductive properties of CPs depend on many intrinsic and extrinsic parameters such as, charge carrier density, delocalization lengthscale of *π*–electrons and structural defects, intra‐and inter‐chain interactions, interaction of the charge carriers with other charges (e.g., (counter)ions, static charge) and dipoles (either from polarization of the material, water, solvent) or reactive species (oxygen, peroxides, superoxides), morphology, crystallinity, synthesis, and processing parameters.^[^ ^90^, ^91^, ^92^, ^93^ ^]^ The high chemical versatility, the compliant mechanical properties, the compatibility with a large variety of cell type, the low impedance over a wider frequency bandwidth compared to metal or metal oxide based coatings, make CPs ideal materials to boost the recording performance of conventional metal‐based neural interfaces^[^ ^83^, ^94^, ^95^, ^96^ ^]^ (see **Box 2**).

A particularly interesting feature for neuroelectronic applications, is the coupled electronic and ionic transport exhibited by a particular subset of CPs, called organic mixed ionic‐electronic conductors (OMIECs).^[^ ^97^, ^98^ ^]^ OMIECs support both electronic charge transport along the *π*‐conjugated polymer chain and ionic transport through the bulk, which accounts for the high (pseudo)capacitance characteristic of OMIEC materials.^[^ ^98^ ^]^ The ability of organic mixed conductors to support ion penetration has enabled the development of electrolyte‐gated organic transistors (EGOTs) capable of amplifying neural signals in situ,^[^ ^20^, ^95^ ^]^ organic ion pumps for on‐demand release of small molecules and drugs,^[^ ^99^, ^100^ ^]^ and neuromorphic devices capable to emulate the characteristic dynamics of synaptic response.^[^ ^101^, ^102^ ^]^ OMIECs can be classified according to their polymeric structure (i.e., heterogeneous blend/co‐polymer or homogenous single‐component polymer) or according to whether or not they intrinsically expose the ionic moiety (or a stabilized charge carrier) on their chains. In the case of non‐intrinsically charged polymers, OMIECs are able to incorporate ions during deposition or directly after exposure to the electrolyte. The fundamental processes that determine the characteristic figure of merit of OMIEC‐based devices are the electronic transport, the ionic transport and the ionic‐electronic coupling. In general, these processes not independent (being their interplay highly complex and still partially unveiled) and are primarily determined by the synthetic design of the material and the processing technique adopted.^[^ ^98^ ^]^ The main advantages of designing OMIEC‐based devices for neural recording are described in **Box 3**.

**Box 3. ECoG Arrays Based on Organic Transistors**

**Figure B3:**
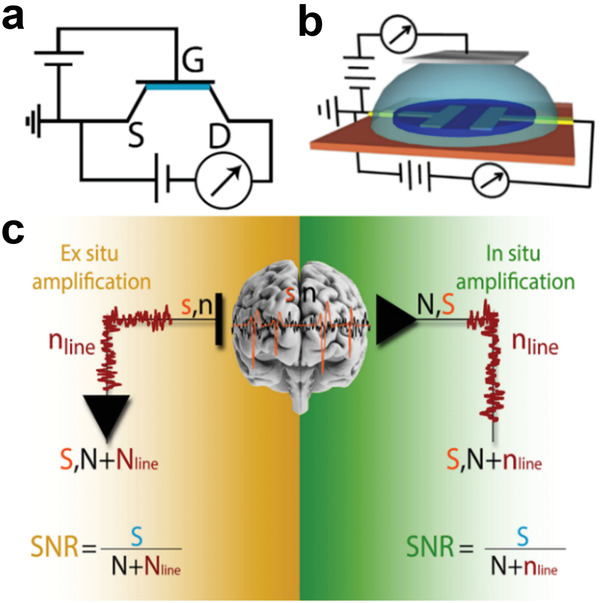
The rationale behind the use of organic transistors for neural recording. a) Electrical connection in an p‐type organic transistor working in depletion mode. S, D, and G represent source, drain, and gate terminals, respectively; b) Schematics and connections layout of EGOT devices; c) In situ versus ex situ amplification by means of transistor‐based neural interfaces. *n* is the biological noise, *s* is the neurophysiological signal, *n*_line_ is the line noise. Capital letters indicate the corresponding amplified signals.

To overcome the inherent limitation of higher noise subsequent to miniaturization, transistor‐based architectures capable of providing in situ amplification of ECoG and μECoG signals represent an emerging alternative of extremely high interest. The key difference between a transistor and an electrode is that the former is an active device, forming a circuit that acts as a voltage‐controlled current amplifier. A voltage‐controlled current amplifier transforms voltage modulations at the gate electrode into current modulations at the drain electrode, while amplifying in situ the signal at the same time (**Figure** **B3**).

When electrodes are used for in vivo recording, an ex situ amplification is required, which involves also the line noise amplification. Downstream amplification thus results in increased line noise which affects the SNR. Instead, when transistors are used, an in situ amplification of the neurophysiological signal and biological noise is carried out. The line noise undergoes no amplification in this case, but is simply transferred as it is. The result is a higher SNR. EGOTs, working either in depletion or in enhancement/accumulation mode, feature an organic semiconductive channel contacted with two terminals, termed Source—S—and Drain—D. The channel is directly exposed to an electrolyte solution, which is in contact with a third terminal, termed Gate—G. The conductance of the organic channel is determined by the density of ions in its proximity. In particular, hole conductance in p‐type channels—including PEDOT:X‐based ones—decreases when the density of cations in proximity of the channel increases (depletion mode), and increases when the density of anions in proximity of the channel increases (accumulation mode). Modulation of channel conductance can thus be achieved by modulating the bias at the gate electrode with respect to the grounded source, while a negative bias at the drain allows to drive and measure a hole current in the channel.EGOTs are commonly referred to as electrolyte‐gated organic field‐effect transistors, putting emphasis on the role of the electric field in the electrolyte in determining the channel conductance, or as OECTs, putting emphasis on the electro‐chemical nature of the channel conductance modulation.^[^ ^130^, ^147^, ^172^, ^173^ ^]^ When EGOTs are operated with constant biases at both the drain and the gate with respect to the source, variations of the channel conductance represent an amplified transduction of variations of the (electro‐chemical) potential of the electrolyte. As a consequence, EGOTs are ideal candidates to perform in situ amplification of ionic currents, such as those generated by brain activity. PEDOT:PSS OECTs were used to transduce epileptiform activity in rats, providing increased SNR with respect to conventional surface microelectrodes.^[^ ^95^, ^131^ ^]^ The high transconductance (i.e., the capability to react with high conductance changes to small potential variations) resulting from the mixed ionic‐electronic transport in organic semiconductors, the easy tunability of device properties and the possibility to work in the wet environment avoiding encapsulation layers and to fabricate large‐area devices by printing and additive techniques, all make EGOT technology suitable for translation to neural recording application.^[^ ^86^ ^]^

Despite these promising results, the use of EGOTs for electrophysiological applications is still limited by two factors: i) EGOTs are operated in the common‐source/common‐ground configuration, which implies the application of a bias in the brain; ii) EGOT working principle implies that higher amplification gain results in decreased bandwidth, and vice versa.^[^ ^119^ ^]^ If the former issue can be circumvented by careful device design in order to achieve maximum gain at zero bias^[^ ^174^ ^]^ or by implementing alternative connection schemes,^[^ ^230^ ^]^ the latter is ubiquitous in EGOTs and hard to avoid with materials currently in use. Noteworthy, EGOT devices were also demonstrated to be highly selective and ultra‐sensitive biosensors for the detection and diagnosis of inflammatory cytokines, anti‐drug antibodies, and neurotransmitters in complex matrices.^[^ ^175^, ^176^, ^177^ ^]^ For instance, OECTs based on PEDOT:tosylate and polyallylamine hydrocholaride composites as channel material were used to detect the neurotransmitter acetylcholine in the range 5–125 *µ*M.^[^ ^178^ ^]^ Finally, EGOT analogues operated in a two‐ or three‐terminal architecture with voltage pulses at fixed amplitude and frequency produce an output current response with either increasing or decreasing amplitude depending on the frequency.^[^ ^179^ ^]^ These devices can also mimic the response of neuronal synapses, embodying in one device some of the functions of neural circuitry, such as short‐term plasticity, spike‐timing‐dependent plasticity, long‐term potentiation, and long‐term depression. These properties are attractive for developing artificial synapses in neuromorphic circuits on the one hand, and prosthetic integration into the brain circuitry on the other hand.^[^ ^116^, ^121^, ^137^, ^180^ ^]^ Therefore, EGOT‐based arrays should be envisaged to be endowed with different functionalities, allowing the design of adaptive multimodal neural interfaces capable of on‐site amplification, signal analysis (including data filtering, pattern recognition, and artefact detection), drug release, and sensing of molecules/proteins of interest.

It is also worthwhile mentioning that several CPs, due to their porous structure and high ionic conductivity, exhibit higher charge storage capacity (CSC) and charge injection capacitance limit values compared to metal and metal oxide electrodes,^[^ ^83^ ^]^ which is a desired characteristic in view of neural stimulation applications.^[^ ^33^, ^103^ ^]^ Nevertheless, poor adhesion to noble metals like Pt, PtIr, Au, swelling and conductivity drifting especially under cyclic stress conditions, strongly limited the application of CPs for chronic applications to date.^[^ ^104^, ^105^ ^]^ Noteworthy, it has been recently reported the possibility to guarantee durable adhesion by modifying the surface of the metal electrodes, for example, by increasing the roughness as in the case of nanostructured Pt or gold electrodes, or by growing a adhesion promoter layer of porous iridium oxide (IrOx), capable to establish a stronger bonding with the CP, via formation of a carbide bond.^[^ ^78^, ^80^ ^]^ Furthermore, as stability of the tissue‐electrode interface can be improved by lowering the mechanical mismatch between the electrode material and the neural tissue, soft 3D hydrogels based on conducting polymers have been recently proposed and tested both in vitro and in vivo (see Section 2.3).^[^ ^106^, ^107^, ^108^ ^]^

### 2.2 PEDOT:PSS: The Workhorse Material for Bioelectronics

**Figure 3:**
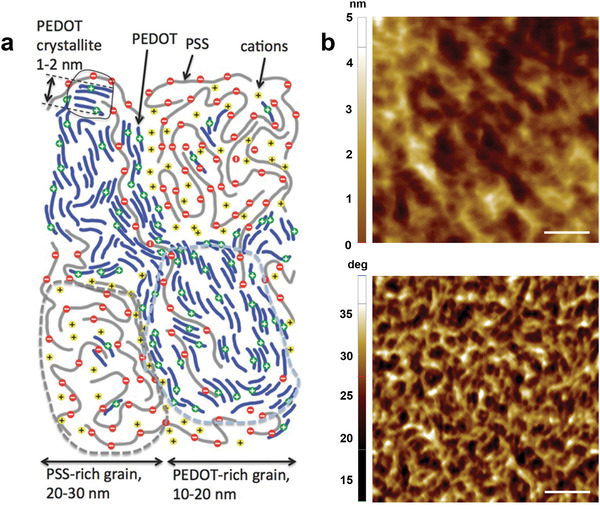
PEDOT:PSS nano‐morphology. a) 2D model encompassing two distinct yet highly interconnected phases consisting of PEDOT‐rich and PSS‐rich domains, as proposed by Volkov and co.Reproduced with permission.^[^ ^117^ ^]^ Copyright 2017, John Wiley and Sons. b) Atomic force microscopy (AFM) topography (i) and phase images of spin‐cast PEDOT:PSS thin films. Scale bar is 100 nm. Darker areas in the phase image (small phase angle deviation) can be associated to soft hygroscopic PSS‐rich areas, whereas brighter areas (larger phase angle deviation) can be associated to “harder” PEDOT‐rich areas (authors’ unpublished data; scale bar is 100 nm).

The prototypical OMIEC material, as well as, the most used CP in bioelectronics, is PEDOT:PSS, a polymer blend of poly(3,4‐ethylenedioxythiophene)(PEDOT) and poly‐(styrene sulfonate) (PSS). The oxidation and polymerization of the EDOT monomer in the presence of the negatively charged PSS counter ion results in an intrinsically highly doped p‐type semiconductor, which behaves as a moderate conductive material. (<1 S cm^−1^).^[^ ^109^ ^]^ Upon mixing with small molecules such as ethylene glycol or dimethyl sulfoxide (known as secondary dopants)^[^ ^110^ ^]^ or treatment with strong acidic solution, PEDOT:PSS increases its conductivity up to 10^3^–10^4^ S cm^−1^.^[^ ^111^, ^112^ ^]^ Because of its mixed ionic/electronic conduction mode, PEDOT:PSS is the first choice as the signal transducing component in bioelectronics devices operated in aqueous electrolytes or hydrogels such as, biosensors and transducers based on organic electro‐chemical transistors (OECTs), low‐impedance multi‐electrode arrays (MEAs) interfaced with the nervous system, up to neuromorphic logic‐based devices and sensors.^[^ ^102^, ^113^, ^114^, ^115^, ^116^ ^]^ The mixed ionic/electronic conductivity in PEDOT:PSS results from the presence of two physically distinct, albeit intimately interpenetrated, phases, one acting as hole‐transport region (PEDOT‐rich phase) and the other as ion transport region (PSS‐rich phase) (**Figure** **3**a).

The lengthscale of multiscale phase separation is from nanometer to mesoscopic length scales and can be observed by atomic force microscopy (Figure 3b).^[^ ^117^ ^]^ The consequent formation of the electrical double layer at the interface between these two domains gives rise to the large effective capacitance that is one of the most desirable properties of PEDOT:PSS. The capacitance of PEDOT:PSS is linearly correlated to its volume for relatively small amount of polymer, typical of those of interest in electrolyte‐gated organic electrochemical transistors (EGOTs) and microelectrodes.^[^ ^118^, ^119^ ^]^ Deviations from this trend at larger polymer volumes were however reported, due for instance to incomplete film hydration^[^ ^120^ ^]^ and saturation of the electroactive area.^[^ ^121^, ^122^, ^123^, ^124^, ^125^ ^]^ When synthesized via chemical route, PEDOT+ and PSS‐ form polymeric particles where positively charged PEDOT chains are surrounded by poly‐anionic PSS shells, the latter providing sufficient stability for PEDOT+ chains to be dispersed in water.^[^ ^126^, ^127^ ^]^ Commercial water‐dispersed PEDOT:PSS formulations offer the great advantage of being a commodity as they allow the easy manufacturing by means of low‐cost additive techniques for a plethora of applications,^[^ ^8^, ^126^, ^128^ ^]^ of antistatic coatings^[^ ^129^ ^]^ and devices such as transistor,^[^ ^130^, ^131^ ^]^ memristors,^[^ ^132^ ^]^ solar cells,^[^ ^133^, ^134^ ^]^ OLEDs,^[^ ^135^ ^]^ supercapacitors,^[^ ^136^ ^]^ and artificial synapses.^[^ ^102^, ^137^ ^]^ Besides, highly conductive PEDOT/PSS films can be grown through the oxidative electrochemical deposition directly on the top of dense neuron‐sized microelectrodes arrays, thus allowing coating in a mask‐less process.^[^ ^16^ ^]^

### 2.3 PEDOT:PSS Hydrogels

CPs form hydrophilic networks, meaning part of the electrode volume will consist of electrolyte absorbed within the material. Thus, already in their pristine form, CPs in electrolytes largely behaves as conducting polymer hydrogels (CPHs). Early work by the Inganäs‐team demonstrated that this hydrogel character could be enhanced by, for example, by merging an ionically cross‐linked hydrogel network of PEDOT:PSS, with electrochemically synthesized PEDOT/PSS.^[^ ^138^ ^]^ The electrochemically formed polymer infiltrated and stabilized the ionically cross‐linked PEDOT:PSS gel, resulting in a highly swollen electrode purely based on PEDOT:PSS. The material had high ionic bulk capacitance and excellent qualities for neural recording electrodes.^[^ ^139^ ^]^ Since then, a large variety of hydrogels and synthesis strategies have been explored, and it is beyond the scope of this text to fully cover this extensive literature. Only a few selected concepts will be brought up here as examples of possibilities relevant for neural interfaces. In particular, in the last few years, exciting new routes have been introduced that allow pure PEDOT:PSS based hydrogels without electrodeposition, thereby enabling the formation of free‐standing blocks of conducting materials. Feig et al. presents a clever protocol where a conducting hydrogel network first is formed by a mix of PEDOT:PSS and an ionic liquid.^[^ ^106^ ^]^ This pre‐formed conducting network is subsequently stabilized by allowing polymerization and cross‐linking of an additional polymer, polyacrylic acid, to form a second network interpenetrating the conductive one. With this strategy both polymers form a truly interpenetrating and homogenous network meaning the mechanical and chemical qualities of the hydrogel truly will be merged with the high electronic and ionic conductivity which signifies PEDOT. Another possibility is presented by Lu et al., who demonstrated that fine‐tuned dehydration of a mixture of dimethyl sulfoxide and PEDOT:PSS dispersions, results in an interconnecting networks of nano‐fibrils. The formed material will be a purely PEDOT:PSS based, and thereby highly conductive, still exhibiting the favorable qualities of true hydrogel.^[^ ^108^ ^]^ The protocols presented by Feig et al. and Lu et al. both are extremely attractive, as they overcome the main limitation of electrodeposition as they allow the formation of 3D blocks of CPHs without the need for a supporting substrate.

For neural interfaces, electrodeposition is nevertheless still the dominant methods, mainly as it has the inherent advantage that the formed material is confined to the conducting parts of the substrate. With electrodeposition CPHs can for instance be formed by allowing the CP to grow through a hydrogel attached to the working electrode. In early work, Kim et al. demonstrated this using an ionically cross‐linked alginate based gel as base, combined with electrodeposition of PEDOT/PSS throughout the preformed gel.^[^ ^140^ ^]^ The authors subsequently demonstrated that such CPH electrodes had excellent in vivo recording performance when used in the auditory cortex.^[^ ^141^ ^]^ A shortcoming of many electrodeposited CPHs is that the CP typically will grow inhomogeneously as the alignment and density of the electrical field will not be the same gel closer to versus further away from the working electrode. Typically, most CP forms closest to the working electrode, with CP structures extending out in a brush‐like pattern though the hydrogel. A true hybrid with homogenous distribution of CP within the hydrogel is more challenging although some tricks have been developed that makes this possible. The group of Green elegantly demonstrated how an interpenetrating growth can be promoted by using a charged hydrogel scaffold, and by depleting any ions in the electrolyte during the electrodeposition.^[^ ^142^ ^]^ This way, the CP will be forced to form along the charged hydrogel backbone, resulting in true integration of the two materials. Green et al. developed this concept using a PVA‐heparin based photocrosslinkable network. This, in addition, made it possible to micro‐pattern the hydrogel scaffold and thus ensure the formed material was confined to the microelectrode underneath. Inspired by this concept, Kleber et al. developed CPHs based on synthetic photo‐crosslinkable charged hydrogels, with the dopant styrene sulphate (SS) immobilized in a backbone of poly(dimethylacrylamide) with methacryloyloxy benzophenone as photo‐reactive group.^[^ ^143^, ^144^ ^]^ The use of a synthetic hydrogel pre‐cursor enabled the use of a C,H insertion crosslinking method, so that cross‐linking of both the hydrogel itself and covalent attachment of the formed hydrogel to a silane treated substrate (iridium oxide), was possible in one and the same step. Subsequent electrodeposition of PEDOT throughout this charged network enabled a true hybrid with high conductivity and electrochemical stability, which also could be successfully micropatterned onto flexible neural devices using standard photolithographic techniques on wafer level.

### 2.4 Beyond PEDOT:PSS

Despite the main advantages and interesting features exhibited by PEDOT:PSS for neuroelectronic applications, some shortcomings have been reported especially with regard to long‐term in vivo applications, including limited adhesion, swelling, and degradation by hydrolysis when exposed to physiological oxidants.^[^ ^145^, ^146^ ^]^ To overcome these problems, composites with carbon nanotubes (CNTs) have been proposed during the last decade, by either doping PEDOT with CNTs during electrodeposition or directly mixed with PEDOT:PSS. The addition of CNTs proved to be a promising approach to prevent material spallation and cracking within the PEDOT:PSS coatings, as well as, PEDOT adhesion during chronic recording and stimulation experiments. At the same time, the excellent electrical properties allow better electrostimulation performance compared to bare PEDOT (see Section 5). Another promising strategy to increase the adhesion of PEDOT coating is based on the physical/chemical modification of the metal or metal oxide underlying electrode surface (see Section 5).

In addition to mechanical issues, the limited charge injection, drift instability, and the high affinity of PEDOT:PSS for divalent cations (which may hinder interactions with other ionic species in extracellular and cerebrospinal fluids, thus limiting the possibility to modulate the input signals from the brain), has led to an extensive exploration of alternative dopants to PSS. To date, several categories of anionic dopants have been investigated, ranging from small ions (e.g., perchlorate or tetrafluoroborate), to acidic molecules (e.g., p‐toluenesulfonic acid, pTS), corticoids (e.g., Dexamethasone) and polymers (e.g., Nafion). Examples of application of PEDOT:X for recording and stimulation experiments are given in Sections 4 and 5.

Noteworthy, alternative materials to PEDOT are currently being investigated for neuroelectronic applications, even not directly as electrode material. For example, 2,7‐dioctyl[1]benzothieno[3,2‐b][1]benzothiophene (C8‐BTBT‐C8) has been used to construct synaptic transistors^[^ ^147^, ^148^ ^]^ and a light‐stimulated devices with both memory and learning capability.^[^ ^149^ ^]^ Light‐stimulated writing/reading neuromorphic memory has also been proposed in light‐gated organic bio‐hybrid transistors (LEGOT) based on TIPS‐pentacene.^[^ ^150^ ^]^ The fused thiophene diketopyrrolopyrrole (FT4‐DPP)‐based conjugated polymer showed small hysteresis whereas the well‐known poly(3‐hexylthiophene‐2,5‐diyl) (P3HT) produced large hysteresis and relatively long memory retention.^[^ ^151^ ^]^ Improved retention time has also been observed with organic semiconductors in which oligoethylene side chains have been inserted for cation coordination.^[^ ^152^ ^]^ In fact, n‐ and p‐type conjugated polymers with oligoethylene glycol side chains appear to be extremely interesting as the electronic/ionic conductivity, specificity toward ions and stiffness can be tuned by appropriately modifying the side chain length and grafting density.^[^ ^152^ ^]^ Furthermore, the incorporation of bioactive functional groups can easily allow bio‐recognition toward molecular signals of interest (stress, inflammation) and its transduction into the electronic properties of the active material.^[^ ^153^ ^]^

## 3 Extracellular Recording with PEDOT:PSS‐Based Neural Interfaces

### 3.1 *μ*ECoG Arrays

**Figure 4:**
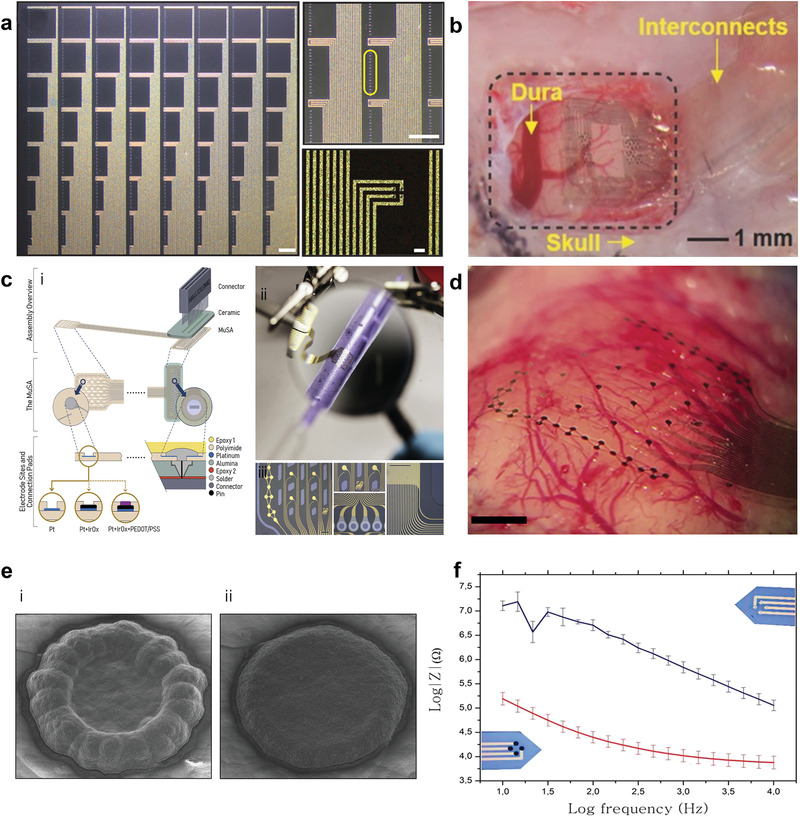
Neural devices based on PEDOT:PSS. a) Photomicrograph of 240‐channel NeuroGrid device (left, scale bar is 1 mm); magnified microscope image of 10 mm^2^ electrodes arranged in 2 × 2 tetrodes, (top‐right, scale bar is 1 mm); high‐density patterning of conducting polymer‐coated electrodes, comprising a single tetrode, (bottom‐right, scale bar is 20 mm). Reproduced with permission.^[^ ^20^ ^]^ Copyright 2016, American Association for the Advancement of Science. b) Neurogrid device placed on the surface of rat somatosensory cortex. Reproduced with permission.^[^ ^161^ ^]^ Copyright 2016, Wiley‐VCH. c) Schematic representation of the fabrication and assembly of a 32 channel MuSA array featuring 10 and 100 µm recording sites made of i) Pt, IrOx, and PEDOT:PSS coatings on the metal electrode; ii) MuSA device wrapped around a pipette; iii) details of the electrode area, connection pads and interconnection tracks of the MuSA array. Scale bars = 200 µm. d) conformable MuSA array on the rat cortex. Scale bar = 1 mm. Reproduced with permission.^[^ ^16^ ^]^ Copyright 2020, Elsevier. e) SEM images of PEDOT:PSS microelectrodes of an intracortical probe obtained by electrochemical deposition using i) 1.1 V and ii) 1.0 V as maximum applied potential; f) impedance plot of PEDOT:PSS coated versus uncoated microelectrode. Reproduced with permission.^[^ ^162^ ^]^ Copyright 2015, Elsevier.

Highly conformable *μ*ECoG arrays featuring PEDOT:PSS‐coated Au microelectrodes were reported by Khodagholy and coworkers in 2011 (**Table** **3**).^[^ ^159^ ^]^ Due to intrinsic lower impedance, PEDOT:PSS coated microelectrodes outperformed Au microelectrodes in the recording of bicucculine‐triggered sharp‐wave events (i.e., mimicking epileptic spikes). Interestingly, PEDOT‐coated electrodes provided similar recording quality as standard intracortical silicon probes featuring Ir microelectrodes. A few years later, the same research group developed the “Neurogrid,” a flexible array of neuron‐size density PEDOT:PSS coated microelectrodes.^[^ ^19^ ^]^ Neurogrid enabled mapping of action potentials from the surface either of the cortex or the hippocampus in freely moving rats,^[^ ^19^ ^]^ as well as, LFPs and action potential waveforms of individual neurons (spikes) from the cortical surface of human subjects undergoing epilepsy surgery (**Figure** **4**a,b).^[^ ^20^ ^]^ Array conformability to the surface of the brain is an essential prerequisite to obtain a stable and high quality recording^[^ ^16^ ^]^ and record both spikes, as well as, LFPs with *μ*ECoG grids.^[^ ^18^ ^]^ Our groups investigated the relationship among polyimide *μ*ECoG conformability, depression of the rat cortex after implantation, and impedance and recording quality in chronic scenario.^[^ ^16^ ^]^ Highly flexible multi‐species array implants of various thickness (4 µm, 8 µm, 12 µm) and conformability, featuring both 10 and 100 µm size microelectrodes of different materials (Pt, IrOx and PEDOT:PSS) were designed and manufactured (Figure 4c,d). Despite showing similar values during the first weeks from implantation, the impedance of poorly conformable arrays began to significantly increase starting from week 3. Histological analysis revealed that the brain depression caused by poorly conformable arrays was significantly higher when compared to thinner devices. Noteworthy, the peak/mean ratio was higher for the most conformable devices, regardless of the electrode material.^[^ ^16^ ^]^ The possibility to record neural signals with high SNR by arrays of OECTs, was reported for the first time by Khodagholy and Co. in 2013.^[^ ^95^ ^]^The key concept for the adoption of OECTs in place of PEDOT:PSS coated electrodes is in the superlinear amplification of the voltage changes into the OECT channel current (see **Box 3**).^[^ ^160^ ^]^ Highly‐conformable ECoG arrays of PEDOT:PSS‐based OECTs, implanted in the somatosensory cortex of epileptic rat models, enabled the detection of both bicuculline‐induced and spontaneous spikes with larger SNR compared to PEDOT:PSS *μ*ECoG electrodes, as well as, intracortical Ir microelectrodes.^[^ ^95^ ^]^

**Table 3:** PEDOT:PSS‐based interfaces for neural recording

						In vivo		
Year	PEDOT deposition	Device substrate	Recording site size	Underlayer	In vitro EIS @ 1 KHz [Ω]	Model/chronic or acute	Notes	Ref.
ECoG Recording								
2011	SC^a)^	Par. C^b)^	(20 × 20 µm^2^)	Au	‐	Rat/A	Better peak definition of 1–10 and 30 Hz bands compared to Au	^[^ ^159^ ^]^
2013	P	Par. C	‐ 12 × 12 µm^2^ ‐ OECT channel: W: 15 µm, L: 6 µm	Au	‐	Rat/A	SNR (dB): Bicucculine‐ induced model 44 (OECT), 24.2 (microelectrode); GAERS model (7–11 Hz) 52.7 (OECT), 30.2 (microelectrode), 32.0 (Ir penetrating electrodes) GAERS model (4–14 Hz) 22.3 (OECT), 13.5 (microelectrode), 18.2 (Ir rprobe)	^[^ ^95^ ^]^
2015	SC	Par. C	10 µm^2^	Pt/Au	‐	Rat/C	Stable SNR (6–9 dB) during all the experimental time in rats (10 days). Recording of LFPs, spiking activity of layer 1 interuneurons, pyramidal cells, and fast firing deep interneurons	^[^ ^19^ ^]^
2015	SC	Par. C	ME 10 µm^2^	Pt/Au	‐	Human/A	Recording of cortical slow oscillation (beta‐frequency) and spiking activity	^[^ ^19^ ^]^
2016	SC	Par. C	10 µm^2^	Ti/Pt/Au	1 × 10^4^–1 × 10^5^	Human/A	High spatiotemporal resolution of LFPs and single spike activity	^[^ ^20^ ^]^
2017	SC	Par. C	OECT channel: W: 70 µm, L: 20 µm	Au	1 × 10^4^	Rat/A	Mapping of neuronal activation potentials with 1 mm^2^ spatial resolution. SNR: 13 dB	^[^ ^167^ ^]^
2018	SC	Par. C	25 × 25 µm^2^	Au	2.6 × 10^4^	Mouse/A	Contextual recording of LFPs and visualization of correlated calcium activity	^[^ ^165^ ^]^
2018	ED	Par. C	Ø: 20 µm	Au	5.4 × 10^3^ (140 µm), 1.21 × 10^4^ (80 µm), 4.34 × 10^4^ (40 µm), 1.3 × 10^5^ (20 µm)	Mouse/C	Contextual two‐photon Ca++ imaging and electrophysiological visually‐evoked recording. Significant impedance increase during the first day from implantation, then steady for all the experiment (2 weeks) Multiunit activity recording in the 0.3–7 kHz band. No cortex inflammation within 20 days.	^[^ ^166^ ^]^
2018	SC	Par. C	15 × 15 µm^2^	Au	7 × 10^3^	Mouse/A	Electrode impedance not influenced by operation of the μFIP	^[^ ^171^ ^]^
2020	P	Par. C	OECT channel 5 < W < 500 µm 5 < L < 500 µm	Au	‐	Rat/C	High definition recording of LFPs Absence of macroscopic tissue damage, neuronal loss, or gliosis.	^[^ ^131^ ^]^
2020	ED	PI	Ø: 10 and 100 µm	Pt/IrOx	4.4 × 10^5^	Rat/C	In vivo impedance increases for all electrodes in poorly conformable arrays compared to highly conformable ones. Peak/Mean ratio greater in IrOx/PEDOT:PSS electrodes than Pt and IrOx ones. Multi‐unit activity recorded from both IrOx and PEDOT:PSS up to 12 weeks.	^[^ ^16^ ^]^
Intracortical recording								
2011	ED	Teflon	Ø: 25 µm	Pt/Ir	1 × 10^4^ (≈15 t. < bare el.)	Rat/C	Small improvement in SNR over 2 weeks of chronic implantation. Impedance of coated electrodes is steadily lower than that of bare electrodes. Rapid increase of impedance during the first week due to gliosis.	^[^ ^83^ ^]^
2015	ED	Par. C	Ø: 20 µm	Ti/Au	1 × 10^4^ (≈70 t.< bare el)	Mouse/C	Higher SNR compared to bare Au electrodes. Action potential recorded.	^[^ ^162^ ^]^
2018	DC	Par. C	Ø: 15 µm	Au	**‐**	Mouse/A	Contextual recording of seizure‐like events and γ‐Aminobutyric acid (GABA) delivery.	^[^ ^171^ ^]^
2019	P	Par. C	15 × 15 µm^2^	Au	1.5 × 10^4^	Mouse/A	High‐fidelity recordings of single‐unit activities from different cortical layers in the brain. Spontaneous and visual‐evoked spike detection across the array. SNR: 29 dB	^[^ ^163^ ^]^
2020	3D	PDMS	Ø: 15 µm	‐	5 × 10^4^–1.5 × 10^5^	Mouse/C	Continuous recording of neural activities in a freely moving mouse, including LFPs and single spikes	^[^ ^164^ ^]^
2020	P	Par. C	OECT channel 5 < W < 500 µm 5 < L < 500 µm	Au	‐	Rat/A	Detection of action potentials from individual neurons.	^[^ ^131^ ^]^

^a)^ P: Photolithography, SC: Spin coating, ED: Electrochemical deposition, DC: Dip coating, SP: Screen printing, 3D: 3D printing

^b)^ Par. C: Parylene C.

### 3.2 Intracortical Probes

**Figure 5:**
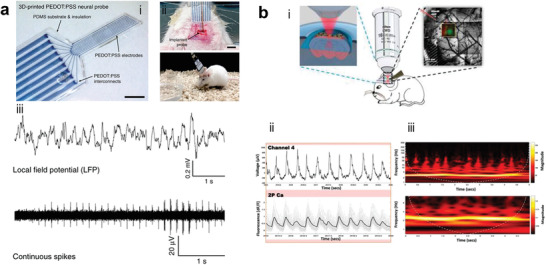
3D printed and multimodal intracortical probes based on PEDOT:PSS. a) Image of an i) all‐PEDOT 3D‐printed soft neural probe, scale bar is 1 mm; ii) images of the implanted probe in mouse cortex; iii) LFPs and spike signals recorded under freely moving conditions. Reproduced with permission.^[^ ^164^ ^]^ Copyright 2020, Springer Nature. b) Layout of the recording setup with 2P‐compatible electrode array, showing the i) infrared laser beam passing through the organic electronic probe placed on the cortex under the objective; ii) electrophysiological local field potential (LFP) recording; and iii) fluorescence variation arising from fluctuations in intracellular calcium concentration. Reproduced with permission.^[^ ^165^ ^]^ Copyright 2018, Society for Neuroscience.

In one of the early works regarding the use of CPs coating for intracortical proves, PEDOT/PSS was electrodeposited on penetrating Pt/Ir microwire arrays in order to perform chronic recording from rat somatosensory cortex.^[^ ^83^ ^]^ PEDOT‐coated electrodes steadily showed lower impedance (−200 Ω) than bare ones, translating into a modest improvement in SNR ratio compared to bare microelectrodes. Castagnola et al. fabricated intracortical Parylene‐C based neural probes featuring gold microelectrodes coated with different PEDOT/PSS layers (Figure 4e,f). The SNR was enhanced both in vitro and in vivo, allowing detection of action potentials and of the noise abatement compared to bare electrodes.^[^ ^162^ ^]^ More recently, the use of highly‐flexible nanomesh PEDOT:PSS arrays enabled the recording of spontaneous and evoked single‐unit activity of neurons across layers of the mouse visual cortex.^[^ ^163^ ^]^ Due to the extremely softness of the parylene‐based probe, a temporary stiffening coating was necessary to facilitate the insertion of the nanomesh arrays into the mouse brain. 3D‐printing has been also investigated as a straightforward method to directly pattern PEDOT:PSS micro‐electrodes on highly‐compliant substrates such as, PDMS (**Figure** **5**a).^[^ ^164^ ^]^ The probe was implanted into the mouse dorsal hippocampus and it continuously recorded neural activities in freely moving mice, including LFPs and action potentials. Very recently, Cea and Co. developed a penetrating array based on innovative electrochemical transistors, named enhancement‐mode internal ion‐gated organic electrochemical transistor (e‐IGT), characterized by high hole mobility, large transconductance, large capacitance, and long‐term operational stability.^[^ ^131^ ^]^ Recording using a common‐source configuration from the somatosensory cortex of a freely moving rat allowed them to detect with high fidelity LFP showing typical spectral features of non‐rapid eye movement sleep, rapid eye movement sleep, and wakefulness, as well as, single‐unit activity.

### 3.3 Multimodal Neural Devices

Additional insights about brain communication networks can be obtained by hybrid approaches where temporally‐resolved electrical recording is coupled with spatially‐resolved optical methods. By leveraging the low impedance of multi‐arrays of PEDOT:PSS electrodes and the optical transparency of a thin parylene substrate, Donahue and Co. simultaneously recorded neural signals and two‐photon calcium imaging from the same location in the cortex of epileptic rats (Figure 5b).^[^ ^165^ ^]^ High correlation between the local variations of fluorescence, due to fluctuations of intracellular calcium concentrations, and the local electrophysiological activity (i.e., LFPs) was demonstrated.

Transparent PEDOT‐based nanomesh MEAs were also developed, allowing both wide‐field epifluorescence and two‐photon Ca^2+^ imaging of visual rat cortex. Visual‐evoked potentials from multi‐unit activity were detected; interestingly, no significant inflammation of the cortex due to the MEA implantation was observed after 20 days.^[^ ^166^ ^]^ Ultra‐flexible and transparent arrays of PEDOT:PSS based OECTs were also developed for high‐resolution mapping *μ*ECoG signals evoked by blue laser light stimulation targeted on the rat's brain surface through a fiber‐guided system.^[^ ^167^ ^]^ For clinical applications, the chance of simultaneously recording neural activity and performing on‐demand drug release is highly attractive, especially for the treatment of drug‐resistant neurological disorders such as epilepsy^[^ ^168^, ^169^ ^]^ (see **Box 4**).

**Box 4. Loco‐Regional Drug Delivery from CP Coatings**

Drug delivery from PEDOT is a loco regional approach aimed to reduce the inflammatory response of the surrounding tissue while preserving optimal electrochemical properties (see **Box 1**). A main advantage of using a drug delivery system based on PEDOT instead of the common microfluidics, for instance, relies on the fact that it enables release on‐demand.^[^ ^202^, ^203^, ^204^ ^]^ Dex and its water‐soluble prodrug dexamethasone sodium phosphate (DEX‐P) are extremely potent anti‐inflammatory and immunosuppressive corticosteroids and were incorporated in neural probes coated with PEDOT to reduce the adverse reaction of the surrounding tissue. For example, Martin and co‐workers electrodeposited PEDOT on a PLGA layer charged with Dex. In this case, the release of the drug was controlled by actuation of PEDOT when subjected to external electrical stimulation.^[^ ^205^ ^]^

**Figure B4:**
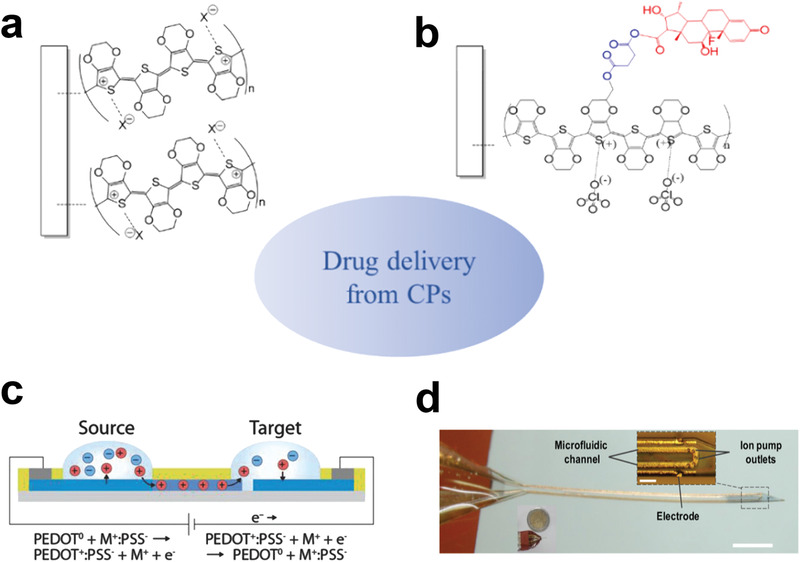
Current approaches for drug delivery from CPs. Incorporation of anionic drugs via a) electrostatic interaction or b) covalent immobilization. c) On‐demand release of cationic drugs from organic electrochemical ion pumps. Reproduced with permission.^[^ ^206^ ^]^ Copyright 2016, Wiley‐VCH. d) On‐demand release of drugs using microfluidic ion pumps. Reproduced with permission.^[^ ^171^ ^]^ Copyright 2018, American Association for the Advancement of Science.

Nevertheless, the typical approach adopted to prepare PEDOT based drug delivery systems consists of the use of the negatively charged drugs, which act as counter ions and can be incorporated during the electrodeposition of the polymeric coating. This is summarized in **Figure** **B4**. The negatively charged dexamethasone‐phosphate (DEX‐P), which is a pro‐drug of Dex, has been extensively incorporated in PEDOT based neural implants.^[^ ^158^, ^184^, ^207^ ^]^ Cui group coated neural microelectrodes with PEDOT electrodeposited in conjunction of oxidized multiwalled carbon nanotubes and Dex‐P. These microeletrodes were used to for stimulation of dorsal root ganglions. They observed that the controlled release of the drug resulted in less neuronal death/damage if compared to uncoated electrodes.^[^ ^158^ ^]^ Following a similar strategy, the same group prepared intracortical neural probes which were tested both in vitro and in vivo, over a period of 11 days in animal models. They found that coated electrodes successfully recorded neural activity throughout the implantation period, setting the stage for the long‐term evaluation of electrically controlled drug release coatings for neural interface applications.^[^ ^158^ ^]^ It was reported that active release of Dex‐P from PEDOT can be controlled over a time frame of up to 4 weeks during in vitro experiments. Indeed, the active drug release should extend for several weeks after implantation in order to mitigate the inflammatory response in chronic neuralexperiments. In addition, a release of 0.5 µg cm^−2^ of Dex corresponds to a concentration of about 1 *µ*M within a 500‐µm radius from the neural microelectrode. This ensures an amount of drug higher than the therapeutic bioactive concentration of Dex, estimated in the range of 0.2 *µ*M.^[^ ^208^ ^]^ Recently, Boehler and co‐workers demonstrated that the active release of Dex‐P can reduce the inflammation around the implanted neural microelectrodes after 12 weeks from implantation. Authors suggested that the combination of flexible probe technology with anti‐inflammatory releasing coatings should be further developed in the future as an effective approach to fabricate long‐term stable chronic neural interfaces.^[^ ^184^ ^]^ Recently, tauroursodeoxycholic acid (TUDCA) was reported to exert neuroprotective function. In particular, this bile acid can reduce microglial and astrocyte cell activation which are normally induced by neural devices implantation.^[^ ^209^, ^210^ ^]^ In light of this, TUDCA was incorporated in PEDOT to prepare an alternative drug delivery system for long term neural recording brain implants.^[^ ^211^ ^]^ A self‐adjusting drug delivery system was proposed by Carli and co‐workers. In this study, Dex was incorporated in PEDOT through covalent bonds, thereby enabling drug release based on chemical bond hydrolysis rather than electrochemical triggers as discussed above. The release of Dex was observed (in vitro) over a period of at least 3 weeks and was accelerated in the presence of hydrolytic enzymes, resembling the biochemical environment of inflamed tissues.^[^ ^212^ ^]^ Recently, new devices enabling on‐demand drug delivery such as, organic electrochemical ion pumps (OEIPs) and Microfluidic Ion Pumps (*μ*FIPs) were proposed. In some case these devices were incorporated in neural probes, allowing simultaneous recording and release of GABA on demand.^[^ ^170^, ^171^ ^]^ Compared to the OEIPs, *μ*FIP significantly reduces the voltage required to deliver ions (drugs) and simplifies re‐loading from the ion reservoir.^[^ ^170^, ^171^ ^]^

In this view, efforts were made to integrate conformable ECoG devices with microfluidic‐based ion pumps (μFIP) that actuate electrophoretic drug delivery.^[^ ^170^, ^171^ ^]^ The μFIP device, endowed with small apertures coated with a PSS‐based exchange membrane for the controlled release of the anti‐epileptic drug (i.e., GABA) upon application of a small voltage, was flanked by 32 PEDOT:PSS‐coated gold microelectrodes for in situ recording of signals with high SNR (Figure 5f).^[^ ^171^ ^]^ Seizure‐like events, induced by local injection of 4‐aminopyridine into the hippocampus of anesthetized mice, triggered the immediate release of GABA, leading to full silencing of additional seizure events.

## 4 Extracellular Recording with Neural Devices Based on PEDOT:X and PPy

**Figure 6:**
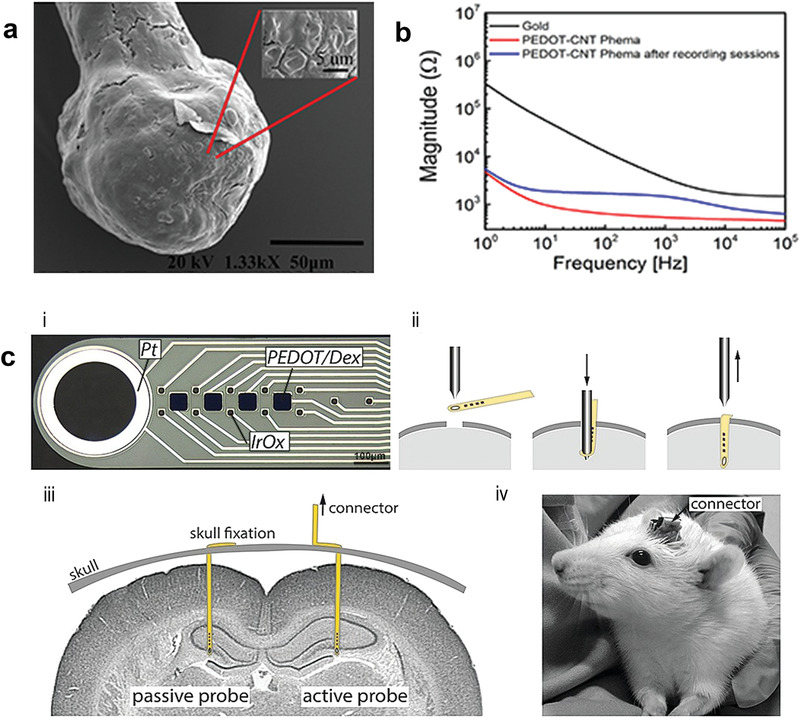
Neural probes based on alternative PEDOT dopants or composites. a) Scanning electron microscopy image of a pHEMA‐coated PEDOT:PSS/CNT microsphere after recordings in rat; b) impedance modulus of a pHEMA‐coated PEDOT:PSS/CNT microsphere before and after recording sessions. Reproduced with permission.^[^ ^183^ ^]^ Copyright 2016, Frontiers in Neuroscience. c) A polyimide neural probe featuring PEDOT/Dex‐coated microelectrodes: i) Optical image of the layout, ii) probe insertion process using an optical fiber as guiding tool, iii) final placement of the intracortical electrodes, iv) image of the implant on site. Reproduced with permission.^[^ ^184^ ^]^ Copyright 2017, Elsevier.

CNTs were added to PEDOT in order to improve its electrical and mechanical properties of the conductive polymer and therefore the performance of epicortical arrays and intracortical probes both in acute and chronic scenarios (a summary can be found in **Table** **4**). For instance, intracortical silicon shafts featuring gold microelectrodes coated with carboxyl‐functionalized MWCNT/PEDOT:PSS composite were used to record acoustically‐evoked single spikes with 2.5 times higher SNR compared to bare electrodes^[^ ^181^ ^]^ or to provide stable recording of visually‐evoked single‐unit and multi‐units activity up to 12 weeks.^[^ ^182^ ^]^ The superior chronic recording performance of PEDOT/CNTs composite electrodes compared to PEDOT were ascribed to the highly‐porous structure and subsequent better integration with the neural tissue. In order to improve the long‐term stability of the neural interface and decrease the mechanical mismatch between device and neural tissue, Castagnola and co. engineered a new kind of neural probe based on PEDOT:PSS/CNT‐coated gold microspheres grown on a Pt wire, surrounded by a soft hydrogel made of poly(2‐hydroxyethyl methacrylate) (pHEMA), added to avoid direct contact between the CNTs and the tissue (**Figure** **6**a).^[^ ^183^ ^]^ The deposition of high surface area PEDOT:PSS/CNT onto the gold nanospheres fourfold lowered the 1 kHz impedance compared to the bare gold (Figure 6b). The slowly resorbable pHEMA hydrogel preserved the electrochemical performance and high quality recording of action potential from the PEDOT:PSS/CNT coated devices during both acute and chronic experiment on rat model. Only a minor occurrence of the inflammatory tissue reaction was observed.^[^ ^183^ ^]^

**Table 4:** Extracellular recording with neural interfaces based on PEDOT:X and on PPy

							In vivo		
Year	CP	Dep. method	Device substr.	Recording site size	Underlayer	In vitro EIS @ 1 KHz	Model/chronic or acute	Notes	Ref.
ECoG									
2015	PEDOT/MWCNT	ED	PI	100 × 100 and 200 × 200 µm^2^	Ti/Au	1 × 10^5^ (≈100 t.< bare el.)	Rat/A	Detection of somatosensory evoked potentials (SEPs) with threefold higher SNR compared to bare electrodes. SNR: 46 ± 13 (spontaneous activity) 77 ± 15, 89 ± 7, 98 ± 30 (whiskers stimuli at 0.8, 1.1, and 1.3 V, resp.)	^[^ ^185^ ^]^
2017	PPy	ED	PDMS	‐	PPy	1 × 10^2^ (≈20 t.< Au el.)	Rat/A	Recording of LFPs in normal rats and epileptic activity (10.5–11.5 Hz) in epileptic rats ECOG	^[^ ^200^ ^]^
2019	PEDOT/Nafion	ED	PI	Ø: 140 µm	Porous Au	1.7 × 10^3^ (≈60 t.< bare. el)	Rat/A	Slightly lower impedance in vitro and similar SNR (8–33 dB) in acute recording of spontaneous LFP activity compared to PEDOT/PSS.	^[^ ^187^ ^]^
Intracortical									
2011	PPy/CNT	ED	PDMS	Ø: 20 µm	Pt/W	9.7 × 10^3^ (≈36 t.< bare el.)	Rat/A	Increases the SNR of spiking signals and of gamma‐range LFPs, without decreasing the SNR in lower frequency LFP bands.	^[^ ^199^ ^]^
2011	PEDOT/TEAPb^)^	ED	Si	Ø: 15 µm	Au	3.7 × 10^5^ (≈25 t.< bare el.)	Rat/C	Foreign body reaction greatly affected activity recording for both coated and uncoated electrodes. However, the low starting impedance of coated sites allowed them to record more quality signals than	^[^ ^155^ ^]^
2013	PEDOT/MWCNT	ED	SOIa^)^	Ø: 30 µm	Au	1.2 × 10^4^ (≈108 t.< bare el.)	Rat/A	Detection of single unit activity following acoustic stimuli including characteristic frequency (CF) and minimum threshold (MT). 2.5 times higher SNR (15 dB) compared to uncoated.	^[^ ^181^ ^]^
2015	PEDOT/MWCNT/DEX	ED	Par. C	Ø: 12 µm	Pt/Ir	27.6 × 10^4^ (≈2 t.< bare el.)	Rat/C	Only spars unit activity recorded. Same SNR (1–4 dB) and noise level between coated and uncoated. Delayed worsening of impedance due to gliosis of coated porous electrodes compared to uncoated ones	^[^ ^158^ ^]^
2015	PEDOT/TFB	ED	Si	Ø: 15 µm	Ir/Au	4 × 10^4^ (≈4 t.< Au el.)	Rat/C	Unit recording up to 12 weeks. SNR of PEDOT:TFB sites (3–25 dB) was higher than that of Au sites only during the first 2 weeks. Impedance in vivo (@1 kHz) significantly increased during the first week likely due to microglia activation and probe insulation.	^[^ ^186^ ^]^
2016	PEDOT/MWCNT	ED	Pt	Ø: 50 µm	Pt/Au	PEDOT:PSS/CNT (1.0 ± 0.2)	Rat/A&C	Similar impedance in vitro and SNR of pHEMA coated and uncoated probe. Detection of action potentials up to 28 days. Limited occurrence of inflammatory response, no obvious neuron loss. SNR ≈ 1.7 dB	^[^ ^183^ ^]^
2016	PEDOT/CNT	ED	Si	Ø: 18 µm	Au	9 × 10^4^ (≈90 t.< bare el.; 1.1 t. < PEDOT:PSS el.)	Mouse/C	Visually evoked single‐unit (SU) and multi‐units (MU) activity invariantly recorded up to 12 weeks with PEDOT/CNTs microelectrodes. Rapid worsening of the recorded signals with PEDOT:PSS microelectrodes. Rapid impedance increase during the first 3 months from surgery.	^[^ ^182^ ^]^
2017	PEDOT/Dex	ED	PI	15 × 15 µm^2^ and 50 × 50 µm^2^	Pt/IrOx	**‐**	Rat/C	Similar LFPs recording and impedance in vivo as PEDOT:PSS electrodes. Low degree of inflammation. Probes exposed to active drug release protocols did have neurons closer to the electrode sites compared to controls.	^[^ ^184^ ^]^
2018	PEDOT/ox‐SWCNHs‐ox‐MWCNTs	ED	PI	Ø: 60 µm	Au	.8 ± 0.4 (CNHs) 3.5 ± 0.4	Rat/A	SEP and spontaneous activity recorder. No differences between CNHs (SNR: 2.0 ± 1.5 dB) and CNTs (SNR: 2.1 ± 1.2 dB).	^[^ ^77^ ^]^
2020	PEDOT/pTS	ED	C	Ø: 6.8 µm	C	1.9 × 10^4^ (≈205 t.< bare el.)	Rat/A&C	PEDOT:pTS electrodes exhibited a decrease in recording yield and an increase in 1 kHz impedance. Negligible tissue damage due to extra small thickness of the carbon fiber.	^[^ ^201^ ^]^

^a)^ Si on insulator

^b)^ Tetraethyleammonium perchlorate.

SEPs and spontaneous activity acute recordings were recently recorded from the rat cortex *μ*ECoG arrays featuring PEDOT:PSS co‐deposited with oxidized single walled carbon nanohorns or oxidized multi walled carbon nanotubes.^[^ ^77^ ^]^ In another study, high quality SEPs were detected from the mouse cortex by means of *μ*ECoG arrays featuring relatively large gold microelectrodes (100 and 200 µm) coated with carboxylate MWCNT/PEDOT:PSS composite.^[^ ^185^ ^]^ Due to the large gain in impedance compared to bare electrodes (3.4‐fold in the LFP band, i.e., 1–250 Hz), MWCNT/PEDOT:PSS electrodes were able to record SEP signals with ≈threefold higher SNR compared to bare electrodes.

Ludwig et al. investigated the possibility to record low noise neural activity from rat motor cortex by depositing PEDOT doped with tetraethylammonium perchlorate (PEDOT/TEAP) on gold electrodes of an intracortical Si probe.^[^ ^155^ ^]^ Single activity was detected only from PEDOT‐coated electrodes due to the lower impedance. However, the average number of recorded units for PEDOT sites was highest in the days immediately following surgery and then diminished over the week following surgery due to the progress of the immune response. Similarly, low impedance PEDOT doped with tetrafluoroborate (PEDOT/TFB) electrodeposited on gold microelectrodes of a single‐shank silicon probe, recorded signals from the rat motor cortex with higher SNR compared to bare electrodes only during the first weeks from implantation.^[^ ^186^ ^]^ After 12 weeks of recording, only one third of PEDOT/TFB sites still recorded unit activity. PEDOT/Nafion copolymer has been recently proposed by Carli et al.^[^ ^187^, ^188^ ^]^ Nafion is a linear copolymer derived from tetrafluoroethylene and either perfluorosulfonic acid or perfluorocarboxylic acid monomers.^[^ ^189^, ^190^, ^191^ ^]^ Its biocompatibility has been demonstrated both in vitro and in vivo.^[^ ^192^, ^193^ ^]^ PEDOT/Nafion electrodeposited on porous gold microelectrodes exhibited slightly lower impedance in vitro compared to PEDOT/PSS; this transduced into comparable noise level and SNR when recording in rodent model.^[^ ^187^ ^]^ Boehler and co. investigated the impact on chronic recording and FBR of controlled release of the anti‐inflammatory corticosteroid Dexamethasone (Dex) from flexible neural probes implanted in the rat hippocampus (Figure 6c).^[^ ^184^ ^]^ The drug was released on a weekly basis over 12 weeks of implantation from electrodeposited PEDOT/Dex microelectrodes by applying a cyclic voltammetry signal in fully awake animals. Dex‐functionalized probes provided stable low frequency (between 2 and 15 Hz) LFPs recordings and impedance characteristics over the entire chronic study, similarly to PEDOT/PSS functionalized control probes. Noteworthy, histological evaluation after 12 weeks of implantation revealed an overall low degree of inflammation around all flexible probes whereas electrodes exposed to active drug release protocols exhibited neurons closer to the electrode sites compared to controls.^[^ ^184^ ^]^

Over the last years PPy has been one of the most studied CP thanks to its numerous desirable features like easy oxidation, water solubility, high electrical conductivity, and commercial availability as water dispersion.^[^ ^194^ ^]^ Moreover, PPy film can be synthesized either chemically or electrochemically.^[^ ^195^, ^196^, ^197^ ^]^ In particular, electrochemical oxidation of pyrrole monomers is the most diffuse approach to deposit PPy films on the surface of neural probe microelectrodes.^[^ ^197^, ^198^ ^]^ PPy/MWCNTs composites were developed by Baranauskas and co. to improve the recording performance of PPy‐based electrodes.^[^ ^199^ ^]^ Low impedance PPy/MWCNTs electrodes were implanted in the vibrissa region of rat somatosensory cortex and allowed to record single unit activity, LFPs and multi‐unit activity with an up to fourfold SNR compared to uncoated microelectrodes.^[^ ^199^ ^]^ Qi et al. recently developed a highly‐stretchable, ultra‐soft (Young's modulus ≈450 kPa) PMDS‐based MEAs featuring PPy electrodes.^[^ ^200^ ^]^ A dense layer of PPy nanowires, deposited between the PPy coating and the underlying PDMS by anodic oxidation, was introduced to limit the detachment of PPy from the PDMS substrate by decreasing of the mechanical mismatch between the two components. Once implanted epicortically on the visual cortex of normal and epileptic rats, PPy MEAs succeeded into recording normal rhythms and epileptic EcoG signals.^[^ ^200^ ^]^

## 5 In Vivo Stimulation with PEDOT‐Based Neural Interfaces

PEDOT‐based electrodes have, thanks to their inherent hybrid charge transfer properties, excellent capabilities to cope with the challenge of ion‐electron transduction at the neural interface (see **Box 2**). The metal substrate may inject the current in electronic form into the PEDOT‐film, while the PEDOT‐film transduce this charge to the surrounding electrolyte in the form of ions. Charge transfer at the interface, between CP and electrolyte, in this way differs from that of a metal, as the metal can inject charge via the double layer capacitance and via electrochemical reactions (e.g., oxidation) which will be specific to the material and the electrolyte.^[^ ^103^ ^]^ The surface of the CP will couple capacitively to the electrolyte, just like a metal electrode. However, as the material is semi‐permeable, the internal surface area of the material contributes with a much larger ionic pseudocapacitance contributed by the bulk of the CP film.^[^ ^213^, ^214^, ^215^ ^]^ This bulk may inject or absorb small ions from/to the electrolyte in response to a shift in polarity. Thus, this reaction is not specific to certain reactants but can involve any ions available at the interface that are small enough to penetrate the film. The dynamics of the stimulation pulse, and the diffusion properties of the specific film, will determine to which extent this larger bulk pseudo‐capacitance can contribute to charge transfer. With the typical short pulses during bi‐phasic stimulation^[^ ^103^ ^]^ only the most superficial layer of the material will in practice be involved in charge injection, which typically results in a charge injection capacity in the range of 2–3 mC cm^−2^ for PEDOT based electrodes.^[^ ^83^, ^215^, ^216^, ^217^ ^]^ With increased pulse durations also deeper layers will contribute to charge injection. For instance, Green et al. quantified this effect showing that the CIC of PEDOT/pTS increased close to linearly, from 2 to 4 mC cm^−2^, when the pulse duration was increased from 200 to 800 µs.^[^ ^215^ ^]^ This large pseudo‐capacitance is also the reason for the enormous discrepancy between reported CSC and CIC of CP films. For instance, in the work of Venkatraman (2011), the CSC for PEDOT was estimated to 120 mC cm^−2^ while the CIC for the same electrodes was limited to 3 mC cm^−2^.^[^ ^83^ ^]^ This can be understood by considering that CSC measures charge exchanged for a sweep at 100 mV s^−1^, meaning a full charge/discharge cycle of a CV takes ≈3 s to complete. CIC accounts for a pulse of a few hundred µs meaning the time allowed for charge and discharge during CV is >7000 longer than for a typical neurostimulation pulse.

The most obvious benefit of using CPs for stimulating applications is the reduced risk for electrochemical side‐reactions as the drastically reduced electrochemical impedance (compared to, e.g., a smooth metal) means a reduction of the voltage needed to drive a certain current. However, relatively few studies exist where CP electrodes are used for micro‐stimulation in vivo. An overview of the available in vivo stimulation studies is provided in **Table** **5**. One likely explanation why the stimulation qualities of CPs are not further exploited could be the frequently reported difficulty in establishing a strong bond between the coating and the electrode substrate.^[^ ^83^, ^218^, ^219^, ^220^ ^]^ Stimulation challenge the interface both at the CP/electrolyte and the CP/metal boundary, meaning reliable adhesion is crucial to have a long lasting benefit of the CP coating. In the last 5 years several teams have contributed solutions to this problem, greatly improving the perspective for using PEDOT also for demanding stimulation applications in vivo.^[^ ^80^, ^221^, ^222^ ^]^ In fact, stability of PEDOT/PSS during stimulation in vitro has currently been demonstrated for > 7 billion bi‐phasic pulses at a fairly high charge injection of 2 mC cm^−2^ without degradation (unpublished data, C Boehler). This finding is encouraging as it demonstrates for that the electrochemical stability of PEDOT/PSS itself is very high, as long as a strong bond is supplied to the underlying interface.

**Table 5:** PEDOT‐based neural devices for in vivo stimulation

						In vivo		
Year	CP dep.	Device substr.	Stimulation electrode size	Underlayer	In vitro CIC at 200 µs	Model/chronic or acute	Notes	Ref
CNS stimulation								
2011	ED	Micro‐wire	Ø: 25 µm and 75 µm	Pt‐Ir	3 mC cm^−2^	Rat/C	2 weeks, rat cortex, cathodic first bi‐phasic stimulation. 10 µA/200 µs and microwire electrodes. Reduced voltage transient compared to PtIr, facilitating safe stimulation.	^[^ ^83^ ^]^
2013	ED	PDMS	Ø: 380 µm	Pt	2 mC cm^−2^	Cat/A	Suprachoroidal stimulation, analyzing the recordings in the visual cortex. Neural responses elicited at charge injection of 76 nC using PEDOT/pTS compared to 85 nC for Pt. At 100 µA, 100 µs charge injection voltage excursion was reduced to 1.5 V compared to 3V for Pt.	^[^ ^215^ ^]^
2017	ED	PI	Ø: 50 µm	Au	2 mC cm^−2^	Rat/A	Typical pulsing parameters 20 µA, 100 µs. Reproducible neural response for 1 mC cm^−2^	^[^ ^217^ ^]^
2017	SA^a)^	PI	Ø: 10 µm	Au	‐	Rat/A	Bi‐phasic pulses using an Mn^2+^ functionalized PEDOT gel coating. The gel covers a large area of the probe, not only the individual microelectrodes.	^[^ ^225^ ^]^
2019	ED	Micro‐wires	325 µm2	Pt‐Ir	‐	Rat/C	Bi‐phasic charged‐balanced at 20 µA, 90 µs (30 µC cm^−2^). Stimulation in subthalamic nucleus over 7 and 15 days with PEDOT:BF_4_	^[^ ^226^ ^]^
2020	ED	PDMS	Ø: 0.31–0.37 mm	Pt	4 mC cm^−2^	Rat/C	Bi‐phasic charged balanced pulses at 200 µA, 300 µs duration (19.3 µC cm^−2^). 5 weeks cochlear stimulation. Comparison of conducting hydrogel (CH) to bare Pt. Some of the CH coating was delaminated and triggered an increased tissue response.	^[^ ^219^ ^]^
PNS (including spinal cord) stimulation								
2015	ED	Micro‐wire	Ø: 100 µm	Au	‐	Rat/A	Intramuscular stimulation, pulse duration 100 µs, amplitudes ranging from 0.1 to 3 mA	^[^ ^227^ ^]^
2015	ED	Micro‐wire	‐	Pt‐Ir	‐	Rat/C	Spinal cord stimulation, 10 days, comparing PEDOT/CNTs with and without additional dexamethasone. Stimulation at 20 µA, 200 µs pulse duration.	^[^ ^216^ ^]^
2017	ED	PDMS	‐	‐	‐	Rat/A	Pulsed stimulation of sciatic nerve, pulse duration in ms range.	^[^ ^200^ ^]^
2019	ED	Carbon fibers	Ø: 7 µm 250 µm cylindrical	C	80 µA, 200 µs	Rat/A	Bi‐phasic stimulation, 10–100 µA, 200 µs, in the spinal cord. Coated fibers were more efficient for stimulation (higher active response). Effect of CP was lost after 6000 pulses.	^[^ ^218^ ^]^
2020	SC	Visco‐plastic PDMS	‐	‐	‐	Rat/C	Voltage pulses up to 1 V (threshold response at 100 mV) to estimate conduction velocity. Total study time, 8 weeks	^[^ ^228^ ^]^

^a)^ SA, self‐assembly.

A major benefit of CPs, which this far largely has been overlooked, is their ability to contribute to more efficient stimulation in the low frequency and DC regime, where metal electrodes typically fail. Dijk et al. explored the relation between PEDOT/PSS film thickness and stimulation properties showing an increase in safe stimulation current with increasing film thickness up to at least 1.3 µm. The characterization was performed for voltage controlled pulses, with duration ranging from 100 µs to 1 ms. Integration of the current, yielding charge, demonstrated an increased charge injected for thicker coatings and also an increase for the longer duration.^[^ ^223^ ^]^ Boehler et al. demonstrated stimulation from PEDOT electrodes for sinusoidal signals in the typical range used for ACS (0.1–4 Hz) and showed that with increasing film thickness of PEDOT/PSS (deposition charge up to 4000 mC cm^−2^, corresponding to an approximate thickness of 28 µm) the increase in maximal safe current was close to linear.^[^ ^224^ ^]^ This correlates well with the findings of Dijk^[^ ^223^ ^]^ and Green^[^ ^215^ ^]^ and demonstrates how the pseudo‐capacitive bulk can be more efficiently exploited by slow signals, even for extremely thick layers. Furthermore, in the same study, DCS was demonstrated over 30 min using the same type of IrOx stabilized PEDOT/PSS. The work on DC stimulation using IrOx:PEDOT/PSS was furthermore extended by Leal et al. demonstrating DCS of cells over several hours.^[^ ^214^ ^]^ As the generation of the direct current only involved electrochemically reversible electrostatic interaction with small ions, the electrodes could be re‐charged with intermittent reversed pulses, offering a completely new concept for biocompatible DCS. In summary CPs do not only outperform most metals with conventional stimulation parameters and charge injection measures, but furthermore can cover a parametric range that typically is not possible to achieve with metal electrodes, namely extremely low frequency and DC.

## 6 Outlook

**Figure 7:**
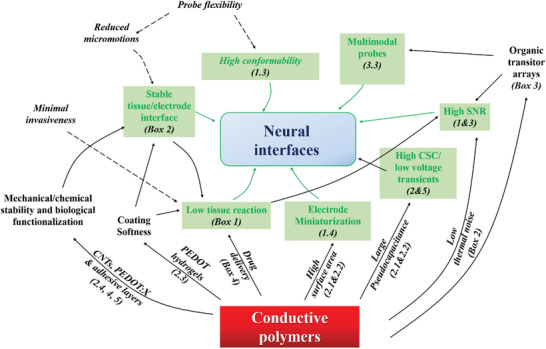
Sketch of the main routes via CPs that can be exploited to build the next generation of neural interfaces for recording and stimulation. Desired properties are in green, while properties not directly derived from the use of CPs but dependent on the device features are in italic. Relative sections or boxes in the text are reported within brackets.

Stable, high‐quality bidirectional communication with neural tissue relies on a number of biotic and abiotic factors that have neither been fully explored nor completely identified. Impaired charge transfer capability at the electrode/electrolyte interface is only one challenge. The structural biocompatibility of neural devices is among the most important factors affecting long‐term performance. The main strategies to address these issues are geared toward engineering the structural aspects of the probe, such as geometry, conformability, and mechanical properties.^[^ ^16^ ^]^ Therefore, design and manufacturing of any neural device has to consider mechanical properties of substrates, interconnection materials, and connectors and their influence on the device properties (e.g., moment of inertia) in interaction with the surrounding tissue. Factors like tethering forces and micromotion strongly influence the integration with the surrounding tissue and the occurrence of chronic gliosis as formidable hurdles to functional probe longevity. All these factors represent a major challenge for materials scientists and engineers and strongly influence the fabrication of long‐lasting neural implantable devices. Interdisciplinary engagement and expertise of material scientists, chemists, electrical, mechanical, and bio‐engineers is needed to converge to designs and performance that overcomes current limitations of chemical and structural biocompatibility. However, the electrode surface is the actual electrochemical and electrical interface between the probe and the brain, and therefore a key component in the design of the most, robust, and lasting devices. From this perspective, conductive polymers represent a very attractive arrow in the bow of materials scientists to improve the recording and stimulation performance of current electrodes. (**Figure** **7**). One of the most obvious reasons for using CPs is the ability to drastically lower the impedance of the metal electrode and hence the thermal noise of the system. This can be particularly relevant in the case of high‐density arrays and small recording sites, which are inherently characterized by high impedance. Nevertheless, how such a pronounced drop in impedance obtained in vitro reflects a real advantage in terms of quality and specificity of the neural signals recorded in vivo, especially during chronic studies, is still a matter of discussion. This can be ascribed to the fact that i) below a certain low impedance value, the shunt loss is minimized regardless of the value of the coating impedance; ii) many factors, including the acute/chronic occurrence of the glial scar, affect the overall recorded signals following still poorly understood pathways, making hard to disentangle the contribution of the CP coating alone to the recorded signal.^[^ ^62^, ^73^, ^229^ ^]^

Importantly, CP coatings offer other advantages besides low impedance, that still make them highly attractive for neuroelectronic applications. For example, CPs represent a softer (albeit not “as soft as brain”) and more suitable tissue‐probe interface than metals or metal oxides. This favors stronger neural cells‐electrode interactions, faster and safer charge transfer across the tissue‐electrode interface and therefore more stable and durable neural interfaces in chronic scenarios. In this context, the recent development of ultra‐soft PEDOT‐based hydrogels allows one to handle 3D conductive interfaces with mechanical properties very close to that of the neural tissue and therefore appears a particularly promising approach.^[^ ^106^, ^107^, ^108^ ^]^ In addition, while transparency of CPs enables the combination of recording of electrophysiological activity with high resolution imaging techniques such as two‐photon imaging,^[^ ^165^ ^]^ CP coatings can be engineered to deliver anti‐inflammatory drugs to decrease FBR^[^ ^184^ ^]^ or anti‐epileptic drugs to prevent the occurring of seizure events.^[^ ^171^ ^]^ Many efforts have been devoted to improving also the long‐term electrical and biological performances of CPs. In particular, mixing or co‐deposition of PEDOT in the presence of pristine or functionalized CNTs has proven to be a preferred approach.^[^ ^199^, ^207^, ^216^ ^]^ However, despite the encouraging results in terms of coating stability and low impedance reported in vitro, only minor improvements were apparently achieved in vivo through this approach. The declination of PEDOT's dopants into functional molecules or polymers and even more the introduction in the field of novel n‐ and p‐type conjugated polymers, appear more as a promising route in order to make a crucial leap.

Besides recording, CPs coatings appear even more effective when used for stimulation purposes. Indeed, their large ionic pseudo‐capacitance allows higher charge injection capacity and lower voltage transients compared to traditional metal electrodes, yielding lower risks of electrochemical side‐reactions during chronic stimulation.^[^ ^83^ ^]^ In addition, CP coatings offer the possibility to work in typical ranges not covered by conventional electrodes, that is, DC and low frequency regimes.^[^ ^214^ ^]^ Shifting the focus away from coatings, CP‐based organic transistors are likely the most reliable approach to make the leap toward high SNR recording in vivo, due to their inherent in situ signal amplification signal. The possibility of recording LFPs, single‐ and multi‐unit activity from the brain surface with the same accuracy of conventional intracortical probes was demonstrated in rats to date.^[^ ^95^ ^]^ Novel propositions addressing the safety “in operando” seem to effectively overcome the issues concerning the applications of additional voltages to the brain surface.^[^ ^230^ ^]^ These reasons, and a robust mature technology also mutated from other organic electronics applications, prompt the consideration that the use of organic transistors for neural recording in humans could be within reach.

## Conflict of Interest

The authors declare no conflict of interest.
